# Influence of total western diet on docosahexaenoic acid suppression of silica-triggered lupus flaring in NZBWF1 mice

**DOI:** 10.1371/journal.pone.0233183

**Published:** 2020-05-15

**Authors:** Kristen N. Gilley, Kathryn A. Wierenga, Preeti S. Chauhuan, James G. Wagner, Ryan P. Lewandowski, Elizbeth A. Ross, A. L. Lock, Jack R. Harkema, Abby D. Benninghoff, James J. Pestka

**Affiliations:** 1 Department of Food Science and Human Nutrition, Michigan State University, East Lansing, Michigan, United States of America; 2 Department of Biochemistry and Molecular Biology, Michigan State University, East Lansing, Michigan, United States of America; 3 Institute for Integrative Toxicology, Michigan State University, East Lansing, Michigan, United States of America; 4 Department of Pathobiology and Diagnostic Investigation, Michigan State University, East Lansing, Michigan, United States of America; 5 Department of Animal Science, Michigan State University, East Lansing, Michigan, United States of America; 6 Department of Animal, Dairy and Veterinary Sciences, School of Veterinary Medicine, Utah State University, Logan, Utah, United States of America; 7 Department of Microbiology and Molecular Genetics, East Lansing, Michigan, United States of America; University of Illinois, UNITED STATES

## Abstract

Lupus is a debilitating multi-organ autoimmune disease clinically typified by periods of flare and remission. Exposing lupus-prone female NZBWF1 mice to crystalline silica (cSiO_2_), a known human autoimmune trigger, mimics flaring by inducing interferon-related gene (IRG) expression, inflammation, ectopic lymphoid structure (ELS) development, and autoantibody production in the lung that collectively accelerate glomerulonephritis. cSiO_2_-triggered flaring in this model can be prevented by supplementing mouse diet with the ω-3 polyunsaturated fatty acid (PUFA) docosahexaenoic acid (DHA). A limitation of previous studies was the use of purified diet that, although optimized for rodent health, does not reflect the high American intake of saturated fatty acid (SFA), ω-6 PUFAs, and total fat. To address this, we employed here a modified Total Western Diet (mTWD) emulating the 50^th^ percentile U.S. macronutrient distribution to discern how DHA supplementation and/or SFA and ω-6 reduction influences cSiO_2_-triggered lupus flaring in female NZBWF1 mice. Six-week-old mice were fed isocaloric experimental diets for 2 wks, intranasally instilled with cSiO_2_ or saline vehicle weekly for 4 wks, and tissues assessed for lupus endpoints 11 wks following cSiO_2_ instillation. In mice fed basal mTWD, cSiO_2_ induced robust IRG expression, proinflammatory cytokine and chemokine elevation, leukocyte infiltration, ELS neogenesis, and autoantibody production in the lung, as well as early kidney nephritis onset compared to vehicle-treated mice fed mTWD. Consumption of mTWD containing DHA at the caloric equivalent to a human dose of 5 g/day dramatically suppressed induction of all lupus-associated endpoints. While decreasing SFA and ω-6 in mTWD modestly inhibited some disease markers, DHA addition to this diet was required for maximal protection against lupus development. Taken together, DHA supplementation at a translationally relevant dose was highly effective in preventing cSiO_2_-triggered lupus flaring in NZBWF1 mice, even against the background of a typical Western diet.

## Introduction

Systemic lupus erythematosus (lupus) is a devastating multi-organ autoimmune disease (AD) that adversely affects 1.5 million Americans, primarily women of child-bearing age [1]. While the genome is a primary predisposing factor for lupus, it is now recognized that environmental exposures over a lifetime can exacerbate or ameliorate disease activity [2, 3]. The initiating step in lupus is loss of tolerance to nuclear self-antigens, resulting in production of autoreactive antibodies and formation of circulating immune complexes [1]. These complexes deposit in the tissues, where they promote activation and infiltration of circulating mononuclear cells leading to organ damage. In the kidney, this manifests as glomerulonephritis that, if left untreated, culminates in end-stage renal failure. Lupus patients typically experience quiescent periods with low disease activity intermittently interrupted by episodes of disease flaring marked by increased symptom severity and active organ damage [4].

Genome-driven mouse models of lupus have been used to elucidate mechanisms of disease pathogenesis and to evaluate efficacy of interventions [5]. Similar to human lupus, female NZBWF1 mice are more likely to develop lupus than their male counterparts [6]. These mice display steady, gradual expansion of autoreactive B and T cells, proinflammatory cytokine and chemokine expression, elevations of autoantibodies, and development of organ damage, thus mimicking the periods of remission in human lupus that precede flaring. Also similar to human lupus, flare-associated disease activity can be initiated and organ damage accelerated in these models by several triggers, including UV exposure [7, 8], epidermal injury [9], and interferon (IFN)-α-expressing adenovirus injection [10–12].

Exposure to the respirable toxicant crystalline silica (cSiO_2_) dust is also a known trigger of lupus and other ADs in humans and animals (reviewed in [13–15]). In lupus-prone female NZBWF1 mice, intranasal instillation with cSiO_2_ mimics flaring by triggering autoimmunity onset three months earlier than controls [16, 17]. When introduced into the lungs, cSiO_2_ initiates chronic sterile inflammation that progresses from local to systemic autoimmunity [18]. Due to their small size (approximately 2 μm), cSiO_2_ particles deposit in the alveoli where alveolar macrophage phagocytose them, ultimately triggering phagolysosome permeabilization. This in turn activates the inflammasome resulting in IL-1 and IL-18 release, as well as cell death by pyroptosis, apoptosis, and necrosis [19, 20]. Because of the slow clearance of cSiO_2_ from the lung, particles released after cell death are again phagocytosed, evoking a vicious cycle of inflammation and cell death. In cSiO_2_-instilled female NZBWF1 mice, we observed the development of ectopic lymphoid structures (ELS) and autoantibodies in bronchoalveolar lavage fluid (BALF) and plasma [Cite Bates 2015, 2018]. Circulating autoantibodies bind their cognate autoantigens resulting in immune complexes that deposit in the kidney, promoting inflammation. Collectively, findings in NZBWF1 mice confirm that, following airway exposure to cSiO_2_, the lung serves as nexus triggering flares of systemic autoimmunity and glomerulonephritis.

Conventional treatments for managing lupus, such a glucocorticoids, have considerable adverse effects, while newer immunotherapies are expensive and benefit only a subpopulation of lupus patients [21]. Therefore, new interventions to prevent or delay lupus flaring are needed. Results of human and animal studies suggest that consumption of ω-3 PUFAs from marine sources can both prevent and resolve inflammation and autoimmune disease, as reviewed previously [22–24]. At the mechanistic level, ω-3 PUFAs modulate immune function by altering; 1) ω-6 incorporation into phospholipids, 2) production of bioactive lipid mediators; 3) intracellular signaling, transcription factor activity, and gene expression; and, 4) membrane structure and ultimately function [22]. These mechanisms may be at play in the protective effects seen in human lupus trials employing ω-3 supplementation [25–27]. Relative to animal studies, we previously assessed the impact of supplementing purified rodent diet with ω-3 PUFA docosahexaenoic acid (DHA) on genome-driven autoimmunity in female NZBWF1 mice [28]. Dietary DHA dose-dependently increased ω-3 PUFA content in the erythrocytes, lungs, kidneys, and spleen while also suppressing the cSiO_2_-triggered the triggered inflammatory response. Remarkably, the decrease in inflammatory response correlated with reduced cSiO_2_-triggered leukocyte infiltration in the kidneys and resultant glomerulonephritis [29–31].

To better parallel human food consumption in rodent feeding studies, the total Western diet (TWD) was formulated to emulate typical American intakes of macro- and micro-nutrients on an energy density basis for rodents [32, 33]. The TWD is based on 50^th^ percentile intakes reported in the Centers for Disease Control National Health and Nutrition Examination Survey (NHANES) for 2007–2008, which were adjusted for differences in caloric intake. Overall, the TWD is not necessarily extreme in the level of any given nutrient, but rather reflects the overall U.S. dietary pattern. The TWD has fewer calories from protein and carbohydrate sources and twice that from fat as compared to the AIN-93G diet, the standard diet fed to rodents in nutritional studies to date. The new TWD diet contains more SFA and monounsaturated fatty acids (MUFAs), less PUFAs, more complex carbohydrates, and twice the level of simple sugars. As such, the TWD better represents typical U.S. nutrition intakes, making it very useful for studies employing animal models of human health and disease [34]. Here, we employed a modified TWD (mTWD) based on U.S. macronutrient intake to assess the impact of DHA supplementation with or without SFA and ω-6 PUFA reduction on cSiO_2_-induced lupus endpoints in NZBWF1 mice. The findings reported herein provide important new insights into the translatability of DHA’s effects on lupus flaring in this novel mouse model.

## Methods

### Animals

Experimental animal procedures were reviewed and approved by Michigan State University’s Institutional Animal Care and Use Committee (AUF # PROTO201800113) in accordance with the guidelines of the National Institute of Health. Female lupus-prone NZBWF1 mice were obtained at 6 wks of age from Jackson Laboratories (Bar Harbor, ME), housed 4 per cage, and allowed free access to feed and water. Only females were included in this study, as the incidence and severity of lupus symptoms is less pronounced in male NZBWF1 mice [35]. Animals were maintained under a 12-hour light/dark cycle with regulated temperature (21–24°C) and humidity (40–55%). Mice were monitored daily by animal facility staff, observed for signs of distress such as rapid heart rate and lethargy, and weighed weekly in case of unexplained weight loss. Veterinary staff was alerted to any potential issues or advised for further monitoring. Following silica installation, animals were under observation for approximately 1 hour immediately following the procedure to identify any adverse events and observed again approximately four hours post-instillation.

### Fatty acid analyses

Fatty acid concentrations in diets and tissues were determined by gas liquid chromatography (GLC) as previously described [30]. Erythrocyte fatty acids were measured by Omega Quant Inc. (Sioux Falls, SD).

### Diet formulation

**Fig 1:**
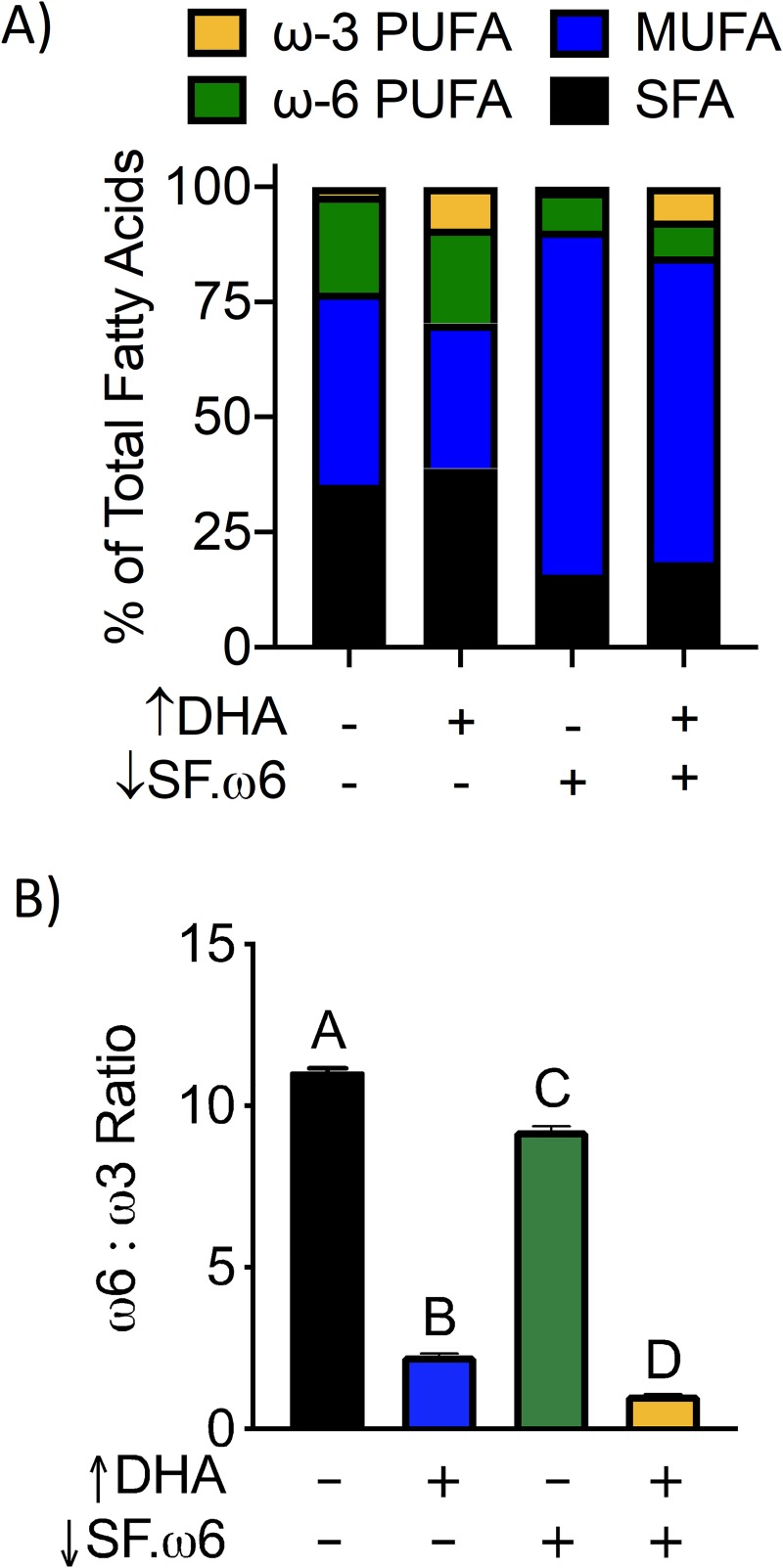
Experimental diets have unique fatty acid compositions. **(A)** DHA supplementation (↑DHA) increased the percentage of total ω-3 PUFAs at the expense of MUFAs. SFAs and ω-6 PUFAs were reduced in the ↓SF.ω6 diet while MUFAs were increased. **(B)** Both ↑ DHA and ↓SF.ω6 diets had lower ω-6:ω-3 ratio than the CON diet. Bars without the same letters differ (p<0.001).

**Table 1** summarizes formulations of the four isocaloric experimental diets used in this study. Since the focus of this study was on macronutrients, a modified version of the TWD (mTWD) was used as a basal diet for all experimental groups, with the modification being that micronutrients were provided by the standard AIN-93G vitamin and mineral mixes. Experimental diets were prepared as follows: 1) control diet (CON) was the basal mTWD with no other alterations; 2) DHA-supplemented diet (↑DHA) was prepared by replacing 30 g/kg of olive oil in the mTWD (composed primarily of the ω-9 monosaturated fatty acid oleic acid) with 30 g/kg DHASCO microalgal oil containing 40% DHA (provided by Dr. Kevin Hadley, Martek Biosciences Corporation Columbia, MD); 3) reduced SFA and ω-6 diet (↓SF.ω6) was prepared by replacing a portion of SFA and ω-6 in the mTWD with olive oil; and 4) ↓SF.ω6 diet supplemented with DHA (↓SF.ω6↑DHA) was prepared by supplementing with DHASCO microalgal oil as indicated above. GLC analysis confirmed the expected changes in amounts of ω-3 PUFAs, ω-6 PUFAs, and SFAs in the four experimental diets (**Table 2**, **Fig 1**). Experimental diets were prepared fresh biweekly, flushed with nitrogen, vacuum sealed, and stored at -20°C until use. Mice were provided fresh diet every day to prevent oxidation of the fatty acids. Palatability was assessed for the first 2 wk to ensure proper consumption.

**Table 1:** Experimental diet formulations.

	Experimental Diet			
	CON	↑DHA	↓SF.ω-6	↓SF.ω-6 ↑DHA
**Ingredient**	*(g/Kg)*			
**Casein**	190.00	190.00	190.00	190.00
**L-Cystine**	2.85	2.85	2.85	2.85
**Corn Starch**	230.00	230.00	230.00	230.00
**Maltodextrin**	70.00	70.00	70.00	70.00
**Sucrose**	256.62	256.62	256.62	256.62
**Corn oil**	16.50	16.50	3.30	3.30
**Soybean oil**	29.40	29.40	5.90	5.90
**Anhydrous milkfat**	36.30	36.30	7.20	7.20
**Lard**	28.00	28.00	5.60	5.60
**Beef tallow**	24.80	24.80	5.00	5.00
**Cholesterol**	0.40	0.40	0.52	0.52
**Cellulose**	30.00	30.00	30.00	30.00
**AIN-93 Mineral Mix**	40.59	40.59	40.59	40.59
**AIN-93 Vitamin Mix**	11.60	11.60	11.60	11.60
**Choline Bitartrate**	2.90	2.90	2.90	2.90
**TBHQ Antioxidant**	0.03	0.03	0.03	0.03
Extra virgin olive oil^**a**^	30.00	0.00	138.00	108.00
DHA-enriched algal oil^**b**^	0.00	30.00	0.00	30.00

^a^Olive oil contained 678 g/kg oleic acid and 84 g/kg linoleic acid, as reported by the USDA, FDC ID 748648

^b^Algal oil contained 395 g/kg DHA and 215 g/kg oleic acid, as reported by manufacturer

**Table 2:** Fatty acid content of experimental diets as determined by GLC.

		Experimental Diet			
		CON	↑DHA	↓SF.ω6	↓SF.ω6↑DHA
Common Name	Chemical Formula	*(% of total fatty acids*, *mean ± SD)*			
Lauric	**C12:0**	0.65 ± 0.01^A^	1.49 ± 0.03^B^	0.14 ± 0.01^C^	0.83 ± 0.01^D^
Myristic	**C14:0**	2.87 ± 0.06^A^	5.54 ± 0.05^B^	0.53 ± 0.01^C^	2.51 ± 0.02^D^
Pentadecanoic	**C15:0**	0.29 ± 0.00^A^	0.30 ± 0.00^A^	0.07 ± 0.00^B^	0.06 ± 0.00^B^
Palmitic	**C16:0**	22.24 ± 0.16^A^	22.63 ± 0.05^B^	10.87 ± 0.06^C^	11.00 ± 0.01^C^
Palmitoleic	**C16:1ω7**	1.15 ± 0.03^A^	1.46 ± 0.02^B^	0.57 ± 0.02^C^	0.84 ± 0.00^D^
Stearic	**C18:0**	8.49± 0.04^A^	8.26 ± 0.03^B^	3.61 ± 0.03^C^	3.37 ± 0.01^D^
Oleic	**C18:1ω9**	39.19 ± 0.38^A^	29.00 ± 0.08^B^	72.63 ± 0.08^C^	64.41 ± 0.03^D^
Linoleic	**C18:2ω6**	21.07 ± 0.21^A^	20.62 ± 0.18^B^	8.57 ± 0.04^C^	7.82 ± 0.04^D^
Arachidic	**C20:0**	0.21 ± 0.03^A^	0.17 ± 0.01^A^	0.29 ± 0.00^B^	0.28 ± 0.01^B^
alpha-Linolenic	**C18:3ω3**	1.90 ± 0.03^A^	1.81 ± 0.04^B^	0.93 ± 0.01^C^	0.87 ± 0.02^C^
Behenic	**C22:0**	0.10 ± 0.01^A^	0.11 ± 0.01^A^	0.10 ± 0.01^A^	0.11 ± 0.00^A^
Lignoceric	**C24:0**	0.04 ± 0.01^A^	0.05 ± 0.01^A^	0.03 ± 0.00^A^	0.05 ± 0.00^A^
Eicosapentaenoic	**C20:5ω3**	0.00 ± 0.00^A^	0.13 ± 0.00^B^	0.00 ± 0.00^A^	0.10 ± 0.01^C^
Docosahexaenoic	**C22:6ω3**	0.00 ± 0.00^A^	7.09 ± 0.20^B^	0.00 ± 0.00^A^	6.34 ± 0.08^C^
Total SFA		35.28 ± 0.21^A^	38.84 ± 0.05^B^	15.80 ± 0.08^C^	18.39 ± 0.02^D^
Total MUFA		41.74 ± 0.36^A^	31.49 ± 0.10^B^	74.70 ± 0.10^C^	66.49 ± 0.01^D^
Total ω-3 PUFA		1.90 ± 0.03^A^	9.04 ± 0.19^B^	0.93 ± 0.01^C^	7.31 ± 0.05^D^
Total ω-6 PUFA		21.07 ± 0.21^A^	20.62 ± 0.18^B^	8.57 ± 0.04^C^	7.82 ± 0.04^D^
ω-6: ω-3 ratio		11.07 ± 0.10^A^	2.28 ± 0.07^B^	9.24 ± 0.13^C^	1.12 ± 0.01^D^

Data presented as mean ± SD. Difference between diets compared by ordinary one-way ANOVA followed by Tukey’s multiple comparison test. Unique letters indicate significant differences between groups (p<0.05)

### cSiO_2_

cSiO_2_ (Min-U-Sil® 5, 1.5–2.0 μm average particle size, U.S. Silica (Mapleton, PA) was acid washed and oven-dried before addition of sterile phosphate buffered saline (PBS) [16]. Stock suspensions were prepared fresh in PBS prior to use, and suspensions were sonicated and vortexed for 1 min before intranasal instillation of each animal.

### Experimental design

**Fig 2:**
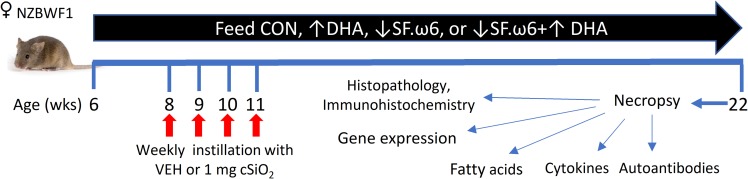
Experimental design. Feeding of experimental diets was begun in 6 wk old female NZBWF1 mice. At 8 wk of age, mice were intranasally instilled with cSiO_2_ once per wk for 4 wk. Body weights were measured weekly, and urine was collected weekly from 18 wk of age onward to monitor the development of proteinuria. Animals were necropsied at 22 wk of age, 11 wk following the final cSiO_2_ instillation. Plasma, BALF, and tissues were collected for analysis.

**Fig 3:**
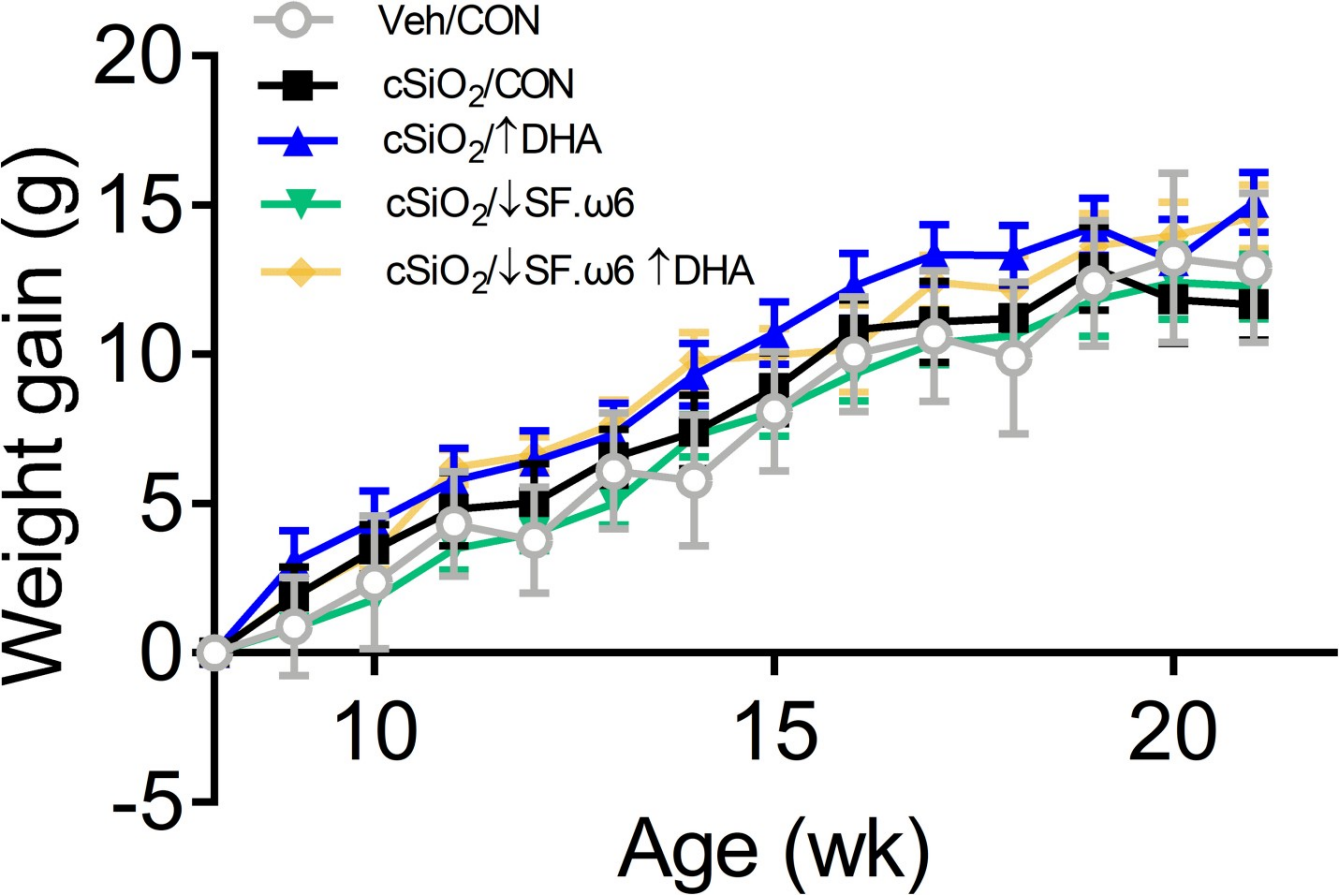
cSiO_2_ instillation and experimental diets did not affect body weight changes over time. Mice were weighed weekly to identify differences in weight gain between diet groups. No significant differences were observed between treatment groups (p < .05).

**Fig 2** depicts the experimental design for this study. Briefly, groups (n = 8) of mice were fed one of the four isocaloric experimental diets beginning at 6 wk of age. 7 mice from the ↓SF.ω6↑DHA diet group were analyzed, as one animal succumbed to an unrelated illness prior to termination of the experiment. After 2 wk, groups of mice were anesthetized with 4% isoflurane and intranasally instilled with 1.0 mg cSiO_2_ in 25 μl PBS or 25 μl PBS vehicle (VEH) as described previously [16]. Body weights were monitored weekly and urine assessed biweekly for proteinuria using clinical dipsticks (Cortez Diagnostics, Calabasas, CA). cSiO_2_ instillation and experimental diets did not affect body weight changes over the course of the study (**Fig 3**) and proteinuria was not detectable. At 22 wk of age (11 wk following the final cSiO_2_ exposure), mice were euthanized by intraperitoneal injection with 56 mg/kg body weight sodium pentobarbital and exsanguination via the abdominal aorta. This time point was selected to capture ectopic lymphoid tissue neogenesis in the lungs following cSiO_2_ exposure prior to and during onset of glomerulonephritis based on previous studies [29, 30]. Blood was collected with heparin-coated syringes and centrifuged at 3500 *x*g for 10 min at 4°C for separation of erythrocytes and plasma, which were then stored at -80°C. BALF was collected from whole lungs as described previously [36] and stored at -80°C for cytokine and autoantibody analysis. The left lung lobe was fixed with 10% (v/v) neutral buffered formalin (Fisher Scientific, Pittsburgh, PA) at constant pressure (30 cm H_2_O) for minimum of 1 h, stored in formalin for 24 h, and then formalin was exchanged to 30% ethanol for long term storage and further processing for histology and immunohistochemistry. The caudal lung lobe was removed, held in RNAlater (Thermo Fisher Scientific, Wilmington, DE) overnight at 4°C, then stored at -80°C for RNA analysis. The right lung, kidney, liver, and spleen were snap-frozen in liquid nitrogen and stored at -80°C for fatty acid analyses.

### IRG expression

Total RNA was extracted from the lung using TriReagent (Sigma Aldrich, St. Louis, MO) per manufacturer’s protocol. Extracted RNA was purified with a Zymo RNA Clean and Concentrator Kit, including DNase digestion to remove any possible DNA contamination (Zymo Research, Irvine, CA, catalog number R1017). Total RNA was quantified using a NanoDrop-100 (Thermo Fisher Scientific) and reverse transcribed to cDNA at 50 ng/ul with a High Capacity cDNA Reverse Transcription Kit (Thermo Fisher Scientific, Waltham, MA). TaqMan Assays were then performed on a SmartChip Real-Time PCR System in technical triplicates for 15 IRGs and 3 housekeeping genes (*Actb*, *Gapdh*, *Hprt*). The IFN score was calculated using 15 IRGs that were significant upregulated in cSiO_2_ treated mice relative to VEH-treated mice (*Ccl7*, *Zbp1*, *Ifi44*, *Ifit1*, *Irf7*, *Isg15*, *Mx1*, *Oas1*, *Oas2*, *Oasl1*, *Psmb8*, *Rsad2*, *Siglec1*, *Ccl8*, *Cxcl10)*, as determined by Student’s t-test for parametric data or Mann-Whitney U test for nonparametric data.

For each gene, the copy number relative to the average expression of *Gapdh*, *Hprt*, *and Actb* was calculated. First, the delta Ct was calculated for each gene by subtracting the mean delta Ct of the two housekeeping genes from the gene of interest. The relative copy number was calculated as previously described [37, 38] using the following equation:

$$RCN=2^{-\Delta Ct}*100$$

Missing values were replaced with ½ the minimum RCN for each gene. For example, the minimum RCN of *Zbp1* was 0.75, thus samples with missing values for *Zbp1* were assigned an RCN ofof 0.37. Then, outliers within each treatment group were identified using robust outlier test (ROUT) with a Q value of 0.05%. After removing outliers, each gene was autoscaled by subtracting the mean expression of each sample and dividing by the standard deviation. The IFN score was calculated by summing the autoscaled expression for each gene within a given sample. All genes were given equal weight.

### Cytokine analyses

Cytokine levels in BALF and plasma were analyzed with Immune Monitoring 48-plex ProcartaPlex Mouse Luminex Bead-based Immunoassay (Thermo Fisher Scientific, catalog number EPX480-20834-901) according to the manufacturer’s protocols at the Michigan State University Flow Cytometry Core using a Luminex 200.

### Autoantibody ELISAs

Autoantibody ELISA kits from Alpha Diagnostic International were utilized as per kit protocols for IgG-specific anti-double-stranded DNA (Alpha Diagnostic, San Antonio, TX, dsDNA, Catalog number 5120), and total anti-nuclear antigens (IgG + IgM + IgA) (ANA/EN, Catalog number 5210) in the BALF and plasma. Samples were read on a FilterMax F3 Multimode plate reader (Molecular Devices, San Jose, CA) at 450 nm.

### BALF cell quantitation and identification

Total viable cell numbers in BALF were determined by Trypan Blue exclusion. Cytological slides from BALF were prepared, allowed to air dry, and stained with Diff-Quick (Fisher Scientific). Differential cell counts for macrophages/monocytes, lymphocytes, neutrophils, and eosinophils in BALF were determined using morphological criteria from 200 total cells on cytological slides. Remaining BALF was centrifuged at 2400 *x*g for 15 min, and supernatant collected and stored at -80°C.

### Lung histopathology

Randomly oriented, serial sections of formalin-fixed left lung lobes were routinely processed and embedded in paraffin. Tissue sections (5 μm) were deparaffinized and stained with hematoxylin and eosin (H&E) for histopathology. Tissues were scored semi-quantitatively by a board-certified veterinary pathologist in a blinded fashion for: (a) presence of lymphoid aggregates within perivascular and peribronchiolar regions; (b) histologically evident ectopic lymphoid tissues; (c) presence of alveolar proteinosis; (d) alveolitis (defined as the increased accumulation in the alveolar parenchyma of neutrophils, lymphocytes, and mononuclear/macrophages); (e) alveolar type II epithelial cell hyperplasia; and (f) mucous cell metaplasia in bronchiolar epithelium. Lungs were individually graded for these lesions as % of total pulmonary tissue examined based on the following criteria: (0) no changes compared to control mice; (1) minimal (<10%); (2) slight (10–25%); (3) moderate (26–50%); (4) severe (51–75%); or (5) very severe (>75%) of total area affected.

### Immunohistochemistry and morphometry of lungs

Immunohistochemistry was performed on formalin-fixed, paraffin embedded, left lung lobe for identification of B and T cell infiltration using anti-CD45R (1:600 rat anti-CD45R monocloncal antibody from Becton Dickinson, Franklin Lakes, NJ, catalog # 550286)and anti-CD3 antibodies (1:250 rabbit anti-CD3 polyconal antibody from Abcam, Cambridge, MA catalog # ab5690), respectively, as described previously [16, 30]. Slides were digitally scanned using a VS110 (Olympus, Hicksville, NY) virtual slide system. At least 100 images were then captured at 20X magnification using systematic random sampling with NewCast software (Visiopharm, Hoersholm, Denmark). Volume densities of CD45R^+^ or CD3^+^ cells in the bronchial and perivascular areas of the lungs were estimated using a point grid over the randomly sampled images with the STEPanizer 1.8 Stereology Tool. The number of points landing directly on the CD45R^+^ or CD3^+^ cells were counted and the volume density or percentage of CD45R^+^ and CD3^+^ per reference area was calculated.

### Kidney histopathology

Fixed kidneys were sectioned, paraffin-embedded, cut and stained with either H&E or Periodic acid-Schiff and hematoxylin (PASH), and evaluated for lupus nephritis by a board-certified veterinary pathologist using a modified International Society of Nephrology/Renal Pathology Lupus Nephritis Classification system [39]. Slide sections were graded as follows: (0) no tubular proteinosis and normal glomeruli; (1) mild tubular proteinosis with multifocal segmental proliferative glomerulonephritis and occasional early glomerular sclerosis and crescent formation; (2) moderate tubular proteinosis with diffuse segmental proliferative glomerulonephritis, early glomerular sclerosis and crescent formation; and (3) marked tubular proteinosis with diffuse global proliferative and sclerosing glomerulonephritis.

### Statistical analysis

Statistical analysis was performed using GraphPad Prism Version 8 (GraphPad Software, La Jolla California USA, www.graphpad.com). First, suspected outliers were verified using the ROUT with a conservative Q value of 1%, meaning that there was <1% chance of excluding a data point as an outlier in error. Data that did not meet the normality assumption as determined by the Shapiro-Wilk test (p<0.01) were analyzed using a the Kruskal-Wallis nonparametric test with Dunn’s post-hoc test for selected multiple comparisons. Data that did not meet the equal variance assumption as determined by the Brown-Forsythe test (p<0.01) were analyzed by the Brown-Forsythe/Welch ANOVA with Dunnett’s T3 post-hoc test for multiple comparisons. Otherwise, data meeting both normality and variance assumptions were analyzed using a standard one-way ANOVA with Sidak’s post-hoc test for multiple comparisons. The groups that were compared were as follows: 1) VEH/CON vs. cSiO_2_/CON; 2) cSiO_2_/CON vs cSiO_2_/↑DHA; 3) cSiO_2_/CON vs. cSiO_2_/↓SF.ω6; 4) cSiO_2_/↑DHA vs. cSiO_2_/↓SF.ω6↑DHA; 5) cSiO_2_/↓SF.ω6 vs. cSiO_2_/↓SF.ω6↑DHA; and, 6) cSiO_2_/CON vs cSiO_2_/↓SF.ω6↑DHA. For samples where every individual in the VEH/CON group was undetectable, a one-sample t-test was performed on the cSiO_2_/CON group to confirm that cSiO_2_-induced changes were significantly different from the limit of quantification. Then, the appropriate statistical test was applied to compare the remaining groups, as described above. In all instances, a significant effect of the diet group was inferred when the adjusted p<0.05.

## Results

### DHA intake increases ω-3 PUFA content in red blood cells and tissues

**Fig 4:**
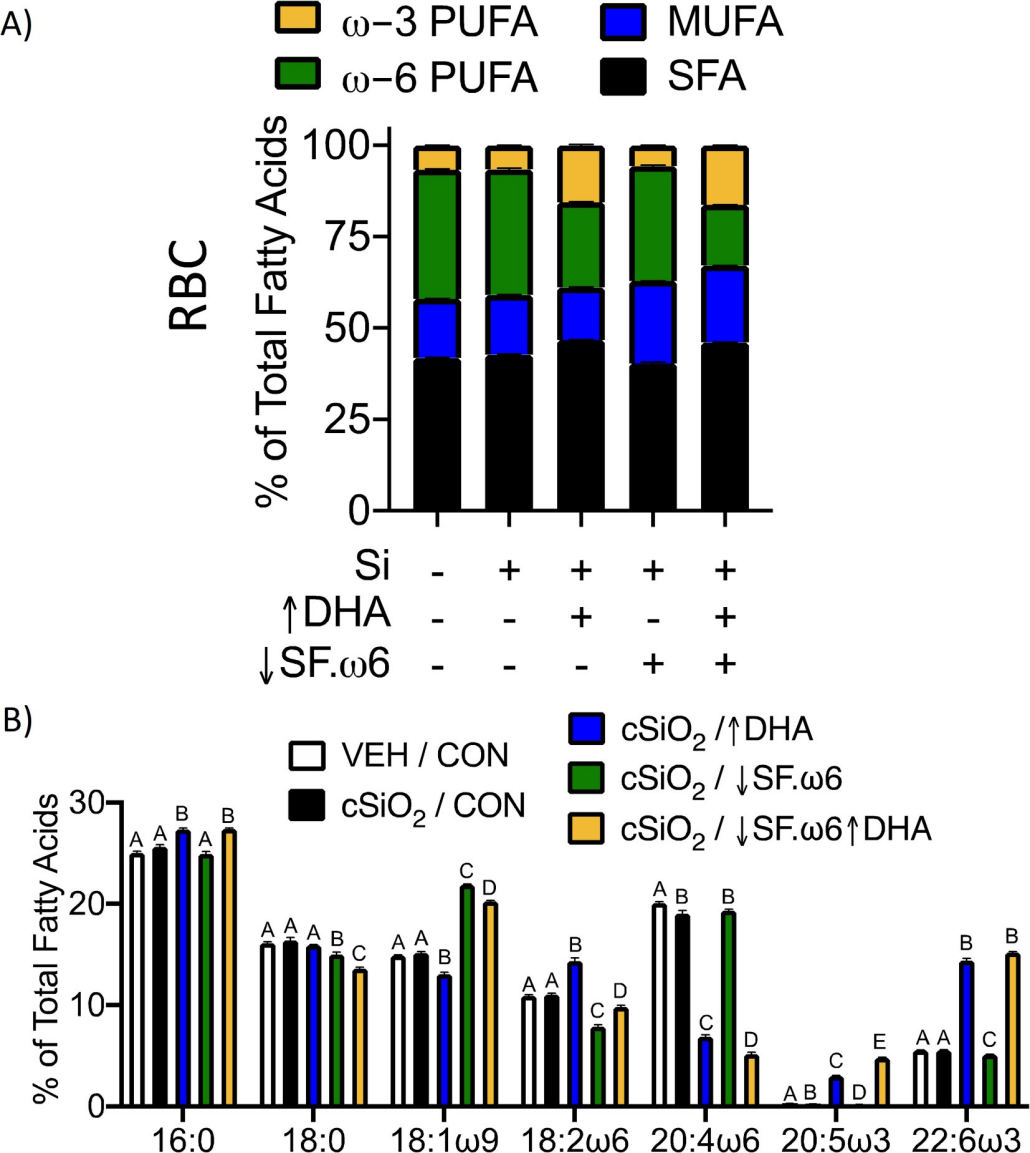
RBC fatty acid composition is influenced by modulation of dietary lipids. Major fatty acid subtypes (**A**) and the 7 most abundant fatty acids (**B**) were compared across treatment groups. Total fatty was determined by GLC and expressed as percent of total. Statistically significant differences in PUFA, MUFA, and SFA are indicated in **Table 3.** Different letters indicate statistically significant differences between treatment groups for individual fatty acids (p<0.05).

**Fig 5:**
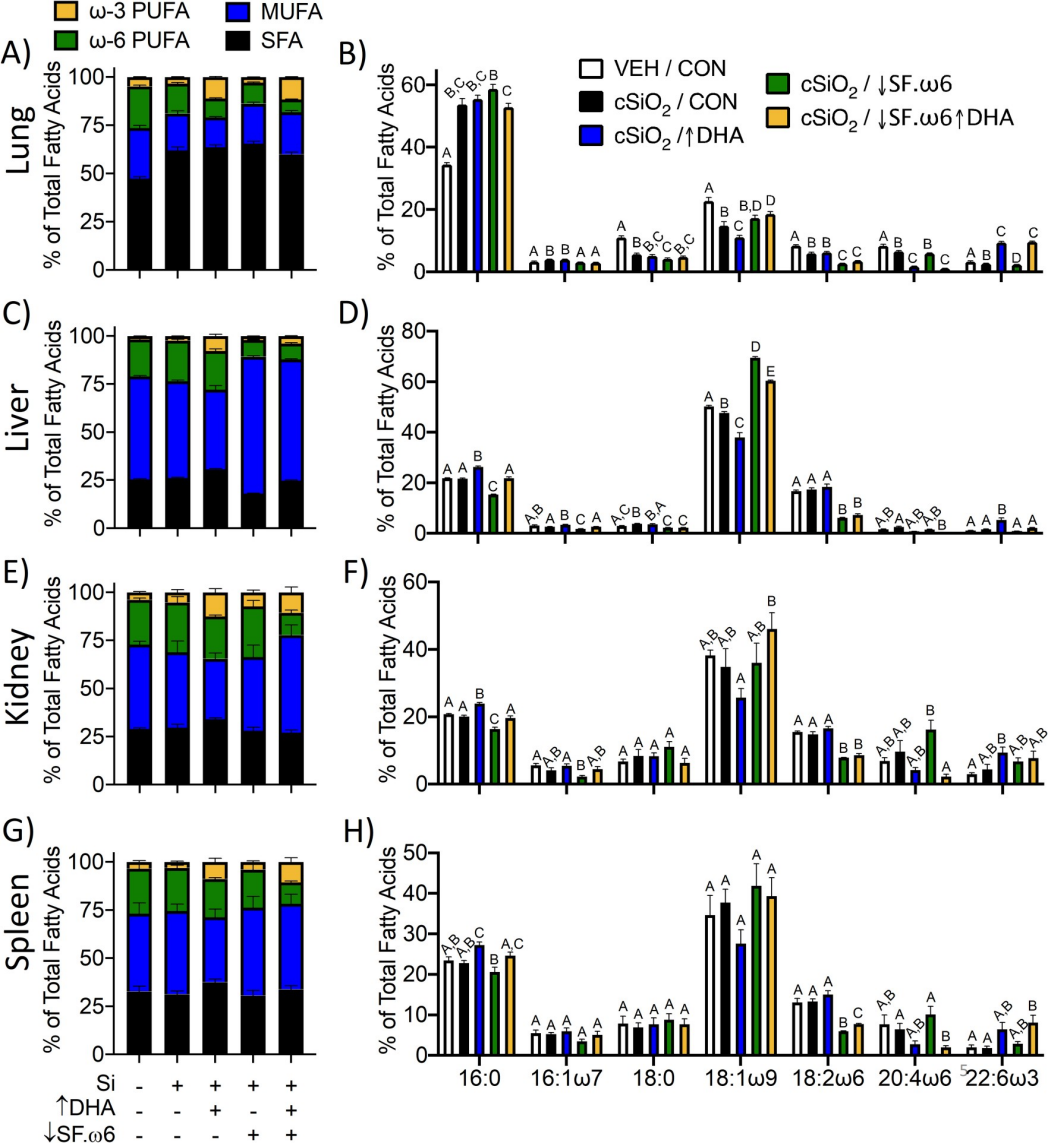
Individual tissues show distinct patterns of fatty acid incorporation. Total fatty acid content was determined by GLC and expressed as percent of total. **(A,C,E,G)** The distribution of SFA, MUFA, and PUFA was compared between tissues. Statistically significant differences are indicated in **S1–S4 Tables. (B,D,F,H,J)** The seven most abundant fatty acids in each tissue were expressed as percent of total. Different letters indicate statistically significant differences between treatment groups for individual fatty acids (p<0.05).

Previous studies have shown that the Omega-3 Index, obtained by measuring DHA and EPA in total red blood cell (RBC) fatty acids, is correlated with the ω-3 PUFA content in other tissues [40–42]. We assessed this, as well as correlations between RBCs and tissues for SFAs, MUFAs, and ω-6 PUFAs. Individual tissues showed unique total FA profiles, with varying degrees of similarity to each other and to RBCs. In general, correlations between RBCs and tissues were higher for ω-3 and ω-6 PUFAs than for SFAs and MUFAs (**S1 Fig**). This is in agreement with previous studies demonstrating high correlations between dietary and tissue levels of fatty acids that cannot be produced endogenously, such as essential ω-3 and ω-6 PUFAs [43–46]. The most consistent trend across all tissues was a concurrent decrease of the major ω-6 PUFA arachidonic acid (ARA) as DHA levels increased (**Figs 4 and 5**).

RBCs had higher levels of total PUFA than the tissues, totaling approximately 40% of RBC fatty acids. The PUFA pool in RBCs was composed primarily of LA, ARA, EPA, and DPA. Total ω-3 incorporation into RBCs was approximately three times greater in mice that consumed ↑DHA or ↓SF.ω6↑DHA diets compared to those fed CON or ↓SF.ω6 diets (**Table 3, Fig 4A and 4B**). Both DHA (22:6ω3) and EPA (20:5ω3) contributed to this increase in total ω-3 PUFA. The increase in EPA observed with DHA supplementation is consistent with published studies performed *in vitro* and *in vivo* [47, 48].

**Table 3:** Red blood cell fatty acid content as determined by GLC.

		VEH / CON			cSiO_2_ / CON			cSiO_2_ /↑DHA			cSiO_2_ / ↓SF.ω6			cSiO_2_ / ↓SF.ω6↑DHA		
Common Name	Chemical Formula	*(% of total fatty acids*, *mean ± SD)*														
Myristic	**C14:0**	0.22	±	0.01^A^	0.23	±	0.03^A^	0.33	±	0.06^B^	0.13	±	0.02^C^	0.22	±	0.02^A^
Palmitic	**C16:0**	24.87	±	0.48^A^	25.47	±	0.67^A^	27.20	±	0.49^B^	24.71	±	0.72^A^	27.05	±	0.38^B^
Palmitelaidic	**C16:1ω7t**	0.14	±	0.01^A^	0.15	±	0.01^A^	0.14	±	0.01^A^	0.07	±	0.01^B^	0.06	±	0.01^B^
Palmitoleic	**C16:1ω7**	0.45	±	0.05^A^	0.45	±	0.06^A^	0.60	±	0.08^B^	0.32	±	0.04^C^	0.41	±	0.03^A^
Stearic	**C18:0**	16.00	±	0.40^A^	16.29	±	0.84^A^	15.83	±	0.18^A^	14.84	±	0.75^B^	13.45	±	0.41^C^
Elaidic	**C18:1ω9t**	0.67	±	0.02^A^	0.69	±	0.04^A^	0.62	±	0.04^B^	0.27	±	0.02^C^	0.24	±	0.02^C^
Oleic	**C18:1ω9**	14.77	±	0.23^A^	15.03	±	0.43^A^	12.99	±	0.55^B^	21.70	±	0.19^C^	20.00	±	0.29^D^
Linoelaidic	**C18:2ω6t**	0.10	±	0.01^A^	0.11	±	0.01^AB^	0.12	±	0.03^AB^	0.08	±	0.01^BC^	0.07	±	0.01^C^
Linoleic	**C18:2ω6**	10.83	±	0.34^A^	10.97	±	0.39^A^	14.30	±	0.78^B^	7.96	±	0.35^C^	9.70	±	0.47^D^
Arachidic	**C20:0**	0.14	±	0.01^A^	0.13	±	0.03^A^	0.13	±	0.03^A^	0.12	±	0.03^A^	0.11	±	0.02^A^
gamma-Linolenic	**C18:3ω6**	0.05	±	0.01^A^	0.06	±	0.01^A^	0.05	±	0.01^AB^	0.05	±	0.00^AB^	0.03	±	0.01^B^
Eicosenoic	**C20:1ω9**	0.27	±	0.01^AC^	0.28	±	0.02^ABC^	0.18	±	0.04^C^	0.49	±	0.06^B^	0.29	±	0.03^AB^
alpha-Linolenic	**C18:3ω3**	0.08	±	0.01^AB^	0.08	±	0.01^B^	0.09	±	0.01^B^	0.04	±	0.01^AC^	0.04	±	0.01^C^
Eicosadienoic	**C20:2ω6**	0.29	±	0.03^A^	0.30	±	0.03^A^	0.30	±	0.03^A^	0.21	±	0.01^B^	0.18	±	0.01^B^
Behenic	**C22:0**	0.13	±	0.04^A^	0.10	±	0.02^B^	0.10	±	0.02^AB^	0.07	±	0.01^B^	0.09	±	0.02^B^
Dihomo-g-linolenic	**C20:3ω6**	1.37	±	0.14^A^	1.26	±	0.09^A^	1.43	±	0.20^A^	1.42	±	0.08^A^	1.24	±	0.14^A^
Arachidonic	**C20:4ω6**	19.92	±	0.49^A^	18.91	±	0.87^B^	6.84	±	0.56^C^	19.14	±	0.45^B^	5.11	±	0.49^D^
Lignoceric	**C24:0**	0.24	±	0.05^A^	0.18	±	0.07^AB^	0.20	±	0.06^AB^	0.14	±	0.03^B^	0.19	±	0.01^AB^
Eicosapentaenoic	**C20:5ω3**	0.30	±	0.03^A^	0.25	±	0.02^B^	2.98	±	0.16^C^	0.20	±	0.02^D^	4.73	±	0.18^E^
Nervonic	**C24:1ω9**	0.20	±	0.04^AC^	0.14	±	0.06^AB^	0.13	±	0.04^B^	0.21	±	0.04^C^	0.26	±	0.02^C^
Docosatetraenoic	**C22:4ω6**	2.23	±	0.09^A^	2.22	±	0.13^A^	0.22	±	0.03^B^	1.98	±	0.15^C^	0.14	±	0.01^B^
Docosapentaenoic ω6	**C22:5ω6**	0.47	±	0.03^A^	0.47	±	0.03^A^	0.05	±	0.01^B^	0.51	±	0.04^A^	0.03	±	0.01^C^
Docosapentaenoic ω3	**C22:5ω3**	0.79	±	0.06^AB^	0.75	±	0.08^A^	0.87	±	0.04^BD^	0.46	±	0.05^C^	0.90	±	0.04^D^
Docosahexaenoic	**C22:6ω3**	5.50	±	0.16^A^	5.50	±	0.19^A^	14.35	±	0.49^B^	5.06	±	0.17^C^	15.00	±	0.28^B^
Total SFA	** **	41.58	±	0.62^AC^	42.39	±	0.72^AB^	43.79	±	0.45^B^	40.01	±	1.13^C^	41.32	±	0.35^AC^
Total MUFA	** **	16.50	±	0.21^A^	16.73	±	0.46^A^	14.65	±	0.57^B^	23.06	±	0.19^C^	21.26	±	0.30^D^
Total ω-3 PUFA	** **	6.67	±	0.16^A^	6.58	±	0.17^A^	18.27	±	0.56^B^	5.76	±	0.21^C^	20.67	±	0.27^D^
Total ω-6 PUFA	** **	35.25	±	0.68^A^	34.30	±	0.89^A^	23.40	±	0.14^B^	31.18	±	0.90^C^	16.49	±	0.37^D^
ω-3 Index	** **	5.80	±	0.15^A^	5.75	±	0.18^A^	17.32	±	0.56^B^	5.27	±	0.17^C^	19.74	±	0.28^D^

Data presented as mean ± SD. Difference between diets compared by ordinary one-way ANOVA followed by Tukey’s multiple comparison. Nonparametric versions of these tests were used when applicable. Unique letters indicate significant differences between groups (p<0.05).

In the RBCs, reducing dietary SFA and ω-6 PUFAs had only a minor impact on SFA content (approximately 4%) and reduced RBC total ω-6 levels by approximately 10%, with the greatest change observed in linoleic acid (LA, 18:2ω6). Alternatively, combining DHA supplementation with ω-6 reduction resulted in decreased RBC ω-6 content by over 50%. The decrease in ω-6 PUFAs with DHA supplementation was largely due to changes in ARA.

The lung was the only organ where a significant change in total FA composition was observed in response to cSiO_2_ **(S1 Table, Fig 5A and 5B)**. Here, cSiO_2_ exposure induced an increase in SFA in the form of palmitic acid (C16:0) from ~30% (similar to RBC levels) to ~50%. Palmitic acid is the fatty acid moiety of dipalmitoylphophatidylcholine (DPPC), a major component of pulmonary surfactant. This increase in palmitic acid appears to be at the expense of stearic acid and oleic acid (OA, C18:1ω9). OA was decreased further by DHA supplementation, but increased slightly in the ↓SF.ω-6 diets. Trends within the PUFA fraction are similar to those observed in the RBCs, with ω-3 PUFAs significantly increased by DHA supplementation.

The liver appeared to be the organ most reflective of dietary fat intake. In the liver, plasma non-esterified fatty acids (NEFAs) obtained from adipose tissue lipolysis or from dietary fatty acids are packaged into lipoproteins to be distributed throughout the body or stored in lipid droplets as triacylglycerides (TAGs) [49–51]. Furthermore, TAGs present in the liver are highly similar to the dietary FA composition, especially when dietary fat remains consistent over time. MUFA levels were greatest in the liver **(S2 Table, Fig 5C and 5D)**, with OA composing ~40% of total liver fatty acids in the CON and ↑DHA diets and ~60% in the ↓SF.ω6 diet and ↓SF.ω6↑DHA diets. This is notable because OA is the major FA in olive oil, which replaced corn oil (composed primarily of LA) in the ↓SF.ω-6 diet. A reduction in SFA and ω-6 PUFAs (mainly in palmitic acid and LA, respectively) was observed in animals fed the ↓SF.ω6 diet. SFA in the liver was comparable to levels in the spleen and kidney (~15%) **(Fig 5E and 5G)**.

The total FA composition of the kidney **(S3 Table, Fig 5E and 5F)** and spleen **(S4 Table, Fig 5G and 5H**) were nearly identical. Unlike RBCs and the liver, there was no clear change in OA in these tissues. Changes in SFA levels across all diets were also minimal. In both tissues, there was a clear decrease in LA with the ↓SF.ω6 diets. The increase in DHA in these tissues was evident, though not as dramatic as observed in the RBC, lung, and liver.

### DHA supplementation suppresses cSiO_2_-induced IRG response in the lungs

**Fig 6:**
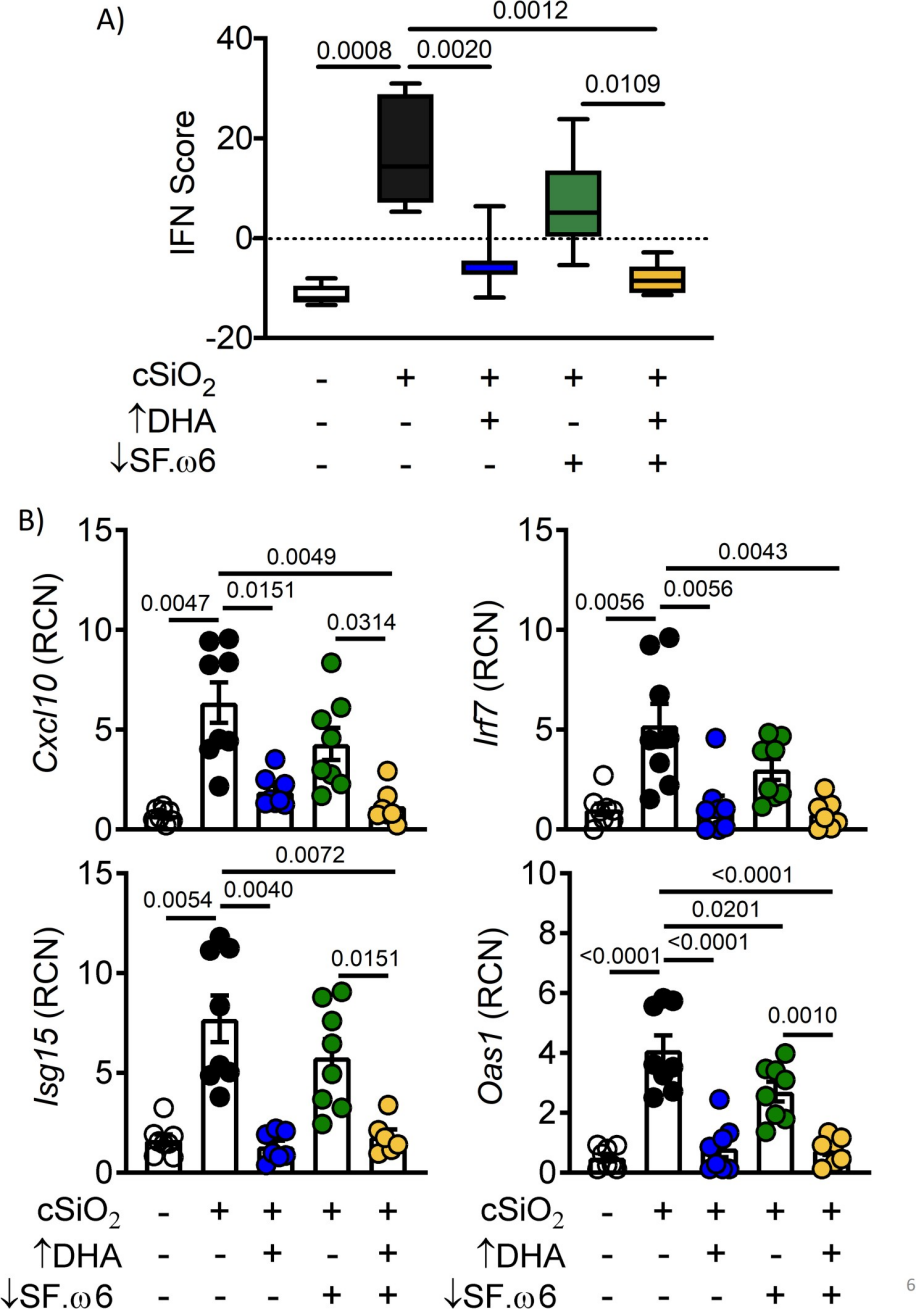
DHA supplementation attenuates cSiO_2_-induced IRG expression in the lungs. **(A)** The IFN score was calculated by combining the auto-scaled expression of 15 differentially expressed IFN-regulated genes (*Ccl7*, *Zbp1*, *Ifi44*, *Ifit1*, *Irf7*, *Isg15*, *Mx1*, *Oas1*, *Oas2*, *Oasl1*, *Psmb8*, *Rsad2*, *Siglec1*, *Ccl8*, *Cxcl10)* for each animal. **(B)** Expression of four representative IRGs. RCN is relative copy number. Values of p<0.1 are shown, with p<0.05 considered statistically significant.

The effects of dietary treatments on cSiO_2_-induced IRG expression in the lung were compared using an IFN score, encompassing 15 genes with known functions in response to IFN signaling. Expression of each of these genes was significantly induced (p<0.05) following cSiO_2_ exposure. The IFN score was calculated for each sample by taking the sum of the autoscaled expression of each gene (see Methods). The IFN score was elevated significantly (p<0.05) in cSiO_2_-instilled mice fed CON but suppressed nearly to baseline in those fed the ↑DHA diet (**Fig 6A**). The IFN score in cSiO_2_-treated mice fed the ↓SF.ω6↑DHA diet was reduced compared to those fed CON or ↓SF.ω6. The mRNA expression profiles for four representative genes, *Irf 7*, *Cxcl9*, *Isg15*, and *Oas1* are illustrative of the expression pattern for individual IRGs (**Fig 6B**).

### DHA intake suppresses cSiO_2_-induced cytokine elevations in the BALF

**Fig 7:**
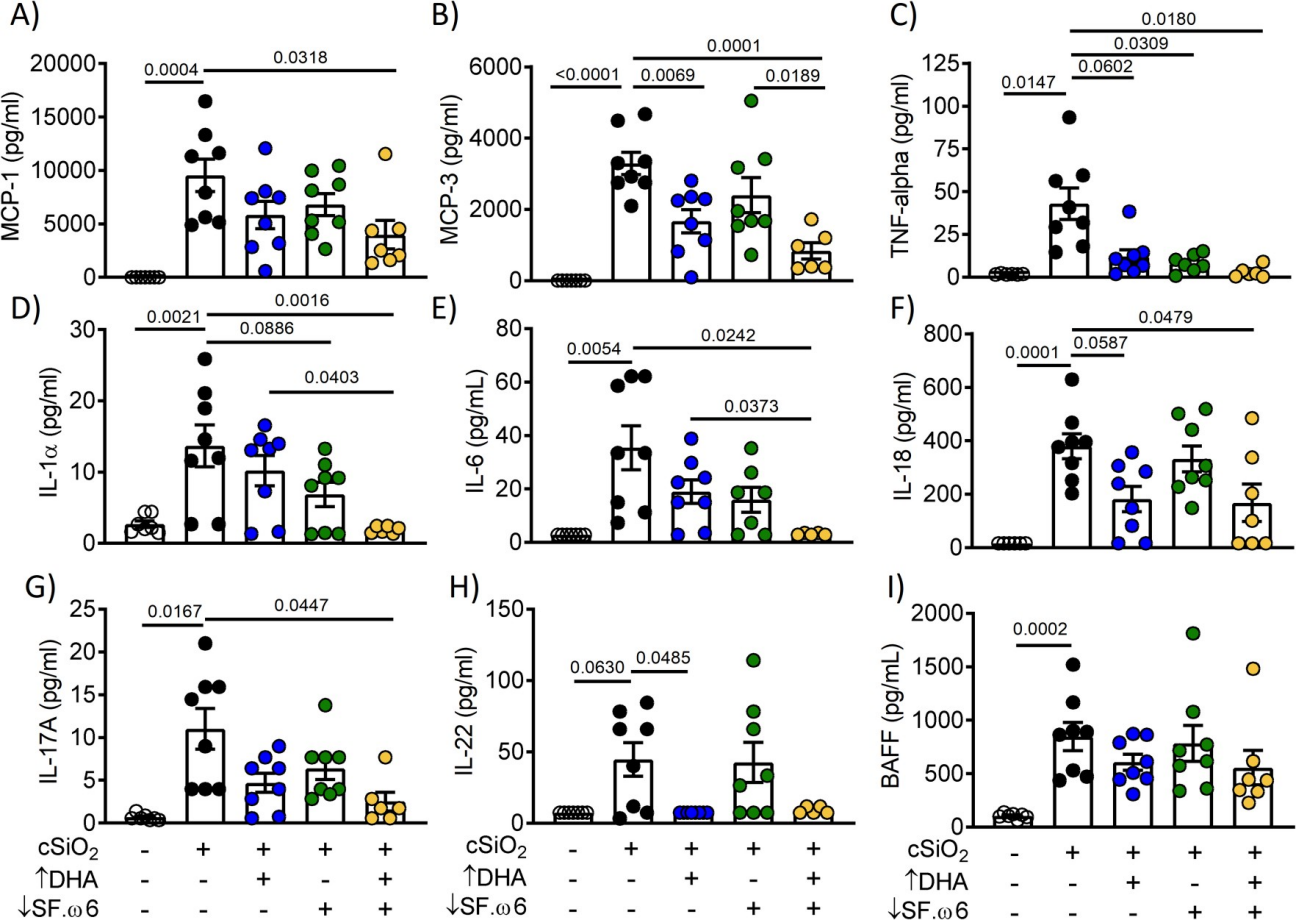
DHA consumption suppresses cSiO_2_-triggered cytokine release. Cytokine levels in the BALF were assessed using antibody bead array. Values of p<0.1 are shown, with p≤0.05 considered statistically significant.

As we have observed in prior studies employing AIN-93G diet [16, 17, 30], cSiO_2_ instillation of mice fed CON diet induced a range of cytokines in BALF that are associated with leukocyte infiltration and the inflammatory response (**Fig 7**). Levels of the monocyte chemoattractant proteins MCP-1 (**Fig 7A**) and MCP-3 (**Fig 7B**) tended to be lower in the BALF of cSiO_2_-treated animals fed ↑DHA, with significant suppression occurring in mice fed ↓SF.ω6↑DHA. Though not all changes were statistically significant, similar observations were made for TNF-α (**Fig 7C**), IL-1α (**Fig 7D**), IL-6 (**Fig 7E**), and IL-18 (**Fig 7F**), which are known for their roles in inflammation and lupus development. Furthermore, T-helper cytokines IL-17A (**Fig 7G**) and IL-22 (**Fig 7H**), which recruit T_H_17 cells and polarize macrophages toward a proinflammatory M1 phenotype, were upregulated by cSiO_2_ in CON-fed mice and downregulated in mice fed ↓SF.ω6↑DHA and ↑DHA, respectively. Finally, while cSiO_2_ instillation induced elevation of B cell activating factor (BAFF) (**Fig 7I**) in the BALF of CON-fed mice, this response was not affected by feeding ↑DHA, ↓SF.ω6, or ↓SF.ω6↑DHA. Overall, DHA supplementation with or without SFA and ω-6 PUFA suppression alleviated induction of many cytokines involved in lupus pathogenesis.

### DHA consumption suppresses cSiO_2_-induced pulmonary immune cell infiltration, including B and T lymphocytes and ELS neogenesis

**Fig 8:**
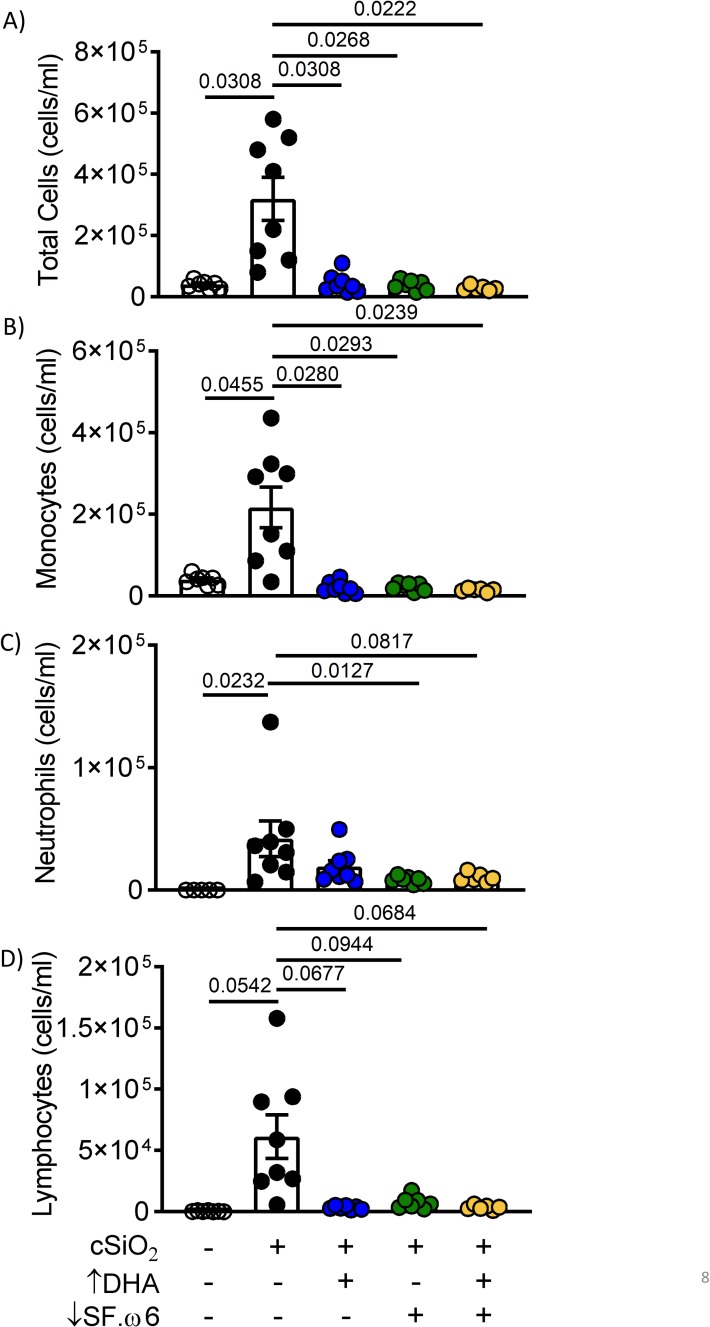
DHA supplementation with SFA and ω-6 PUFA reduction suppress cSiO_2_-induced immune cell accumulation in BALF. BALF was collected at experiment termination and (**A)** total cells, **(B)** monocytes, **(C)** neutrophils, and **(D)** lymphocytes enumerated. Values of p<0.1 are shown, with p<0.05 considered statistically significant.

Further consistent with prior investigations utilizing AIN-93G diet [16, 30], intranasal instillation with cSiO_2_ increased total cells (**Fig 8A**), monocytes (**Fig 8B**), neutrophils (**Fig 8C**), and lymphocytes (**Fig 8D**) in the BALF of mice at experiment termination. Notably, total cell and monocyte accumulation were inhibited in mice fed ↑DHA, ↓SF.ω6, and ↓SF.ω6↑DHA diets. Similar trends were observed with the increase for neutrophils and lymphocytes, though not all were statistically significant.

**Fig 9:**
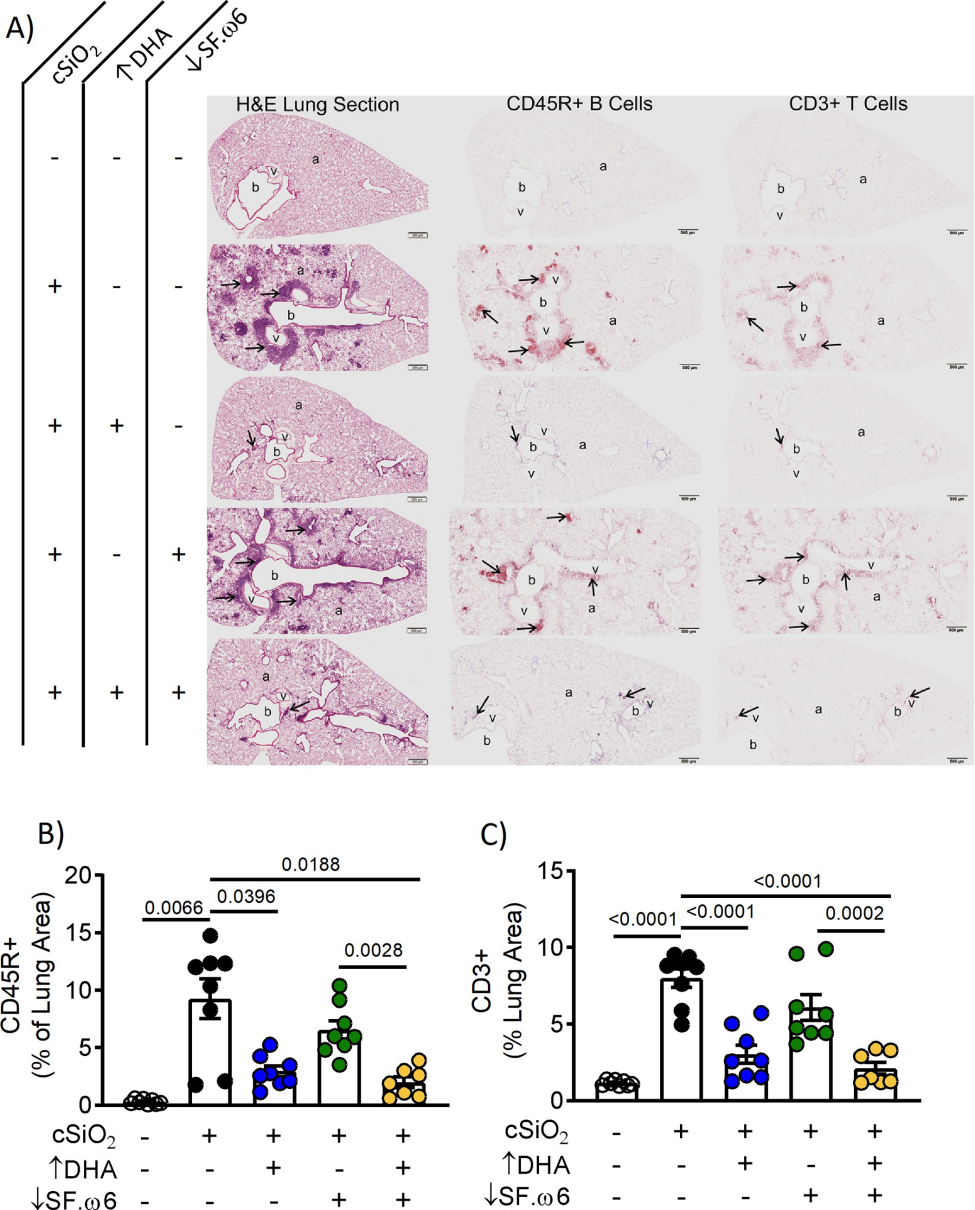
DHA supplementation impedes perivascular and peribronchiolar lymphocyte infiltration, and the neogenesis of ELS. **(A)** Light photomicrographs of tissue sections from the left lung of mice intranasally instilled with crystalline silica (cSiO_2_; +) or saline (VEH alone; -), fed a diet with (+) or without (-) DHA supplementation, and with (+) or without (-) SF.ω6 reduction. Lung sections were stained with H&E (first column), immunohistochemically stained for CD45R^+^ B lymphoid cells (arrows; brown chromagen; second column), or CD3^+^ T lymphoid cells (arrows; brown chromagen; third column). Peribronchiolar and perivascular accumulations of B and T lymphoid cells (ELS; arrows) were present in the lungs of cSiO_2_-exposed mice fed diets without DHA supplementation (second and fourth rows). B or T cell accumulations were not observed in the lung of control mice intranasally instilled with saline and fed CON (row one). Minimal perivascular and peribronchiolar accumulations of B and T lymphoid cells were seen in the lungs of mice intranasally exposed with cSiO_2_ and fed ↑DHA (row three) or ↓SF.ω6↑DHA. (row five). **Abbreviations:** a–alveolar parenchyma, b–bronchiolar airway, v–blood vessel. Morphometric analysis was used to quantitatively determine the volume density of (**B**) CD45R^+^ and (**C**) CD3^+^ area in the measured lung area. Values of p<0.1 are shown, with p<0.05 considered statistically significant.

Histological assessment of H&E-stained lung tissue from cSiO_2_-instilled mice fed CON showed robust peribronchiolar and perivascular leukocytic infiltration (**Fig 9A**). Immunohistochemical staining further indicated cSiO_2_-induced development of ELS in the lung, as evidenced by the organized accumulation of B cells (CD45R^+^) and T cells (CD3^+^) (**Fig 9A**). cSiO_2_-induced cell infiltration and ELS neogenesis were suppressed in mice fed ↑DHA and ↓SF.ω6↑DHA diets, but unlike the observations in BALF, not affected in mice fed the ↓SF.ω6 diet. Morphometric analysis confirmed that cSiO_2_ treatment of mice fed CON diets elicited accumulation of B cells (**Fig 9B**) and T cells (**Fig 9C**) in the lung, further suggestive of ELS neogenesis, and that this response was markedly suppressed in mice fed ↑DHA and ↓SF.ω6↑DHA diets.

### cSiO_2_-induced autoantibody production is attenuated by DHA supplementation

**Fig 10:**
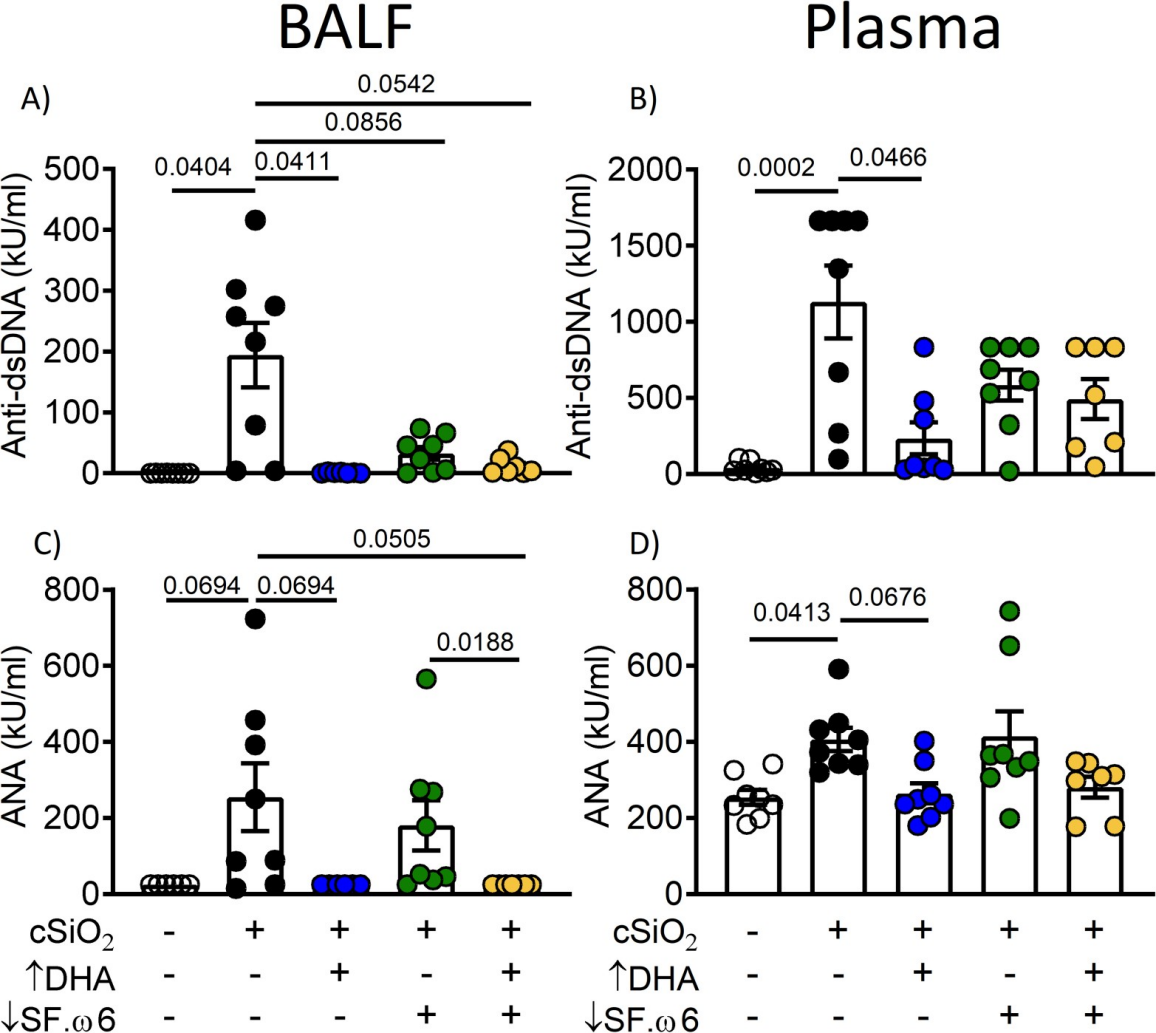
DHA intake suppresses cSiO_2_-induced lupus-associated autoantibodies in the BALF and the plasma. Effects of cSiO_2_ and experimental diets on anti-dsDNA antibodies (**A,B**) and anti-nuclear antibodies (ANA) (**C,D**) in BALF (**A,C**) and plasma (**B,D**). Values of p<0.1 are shown, with p<0.05 considered statistically significant.

Elevations in anti-dsDNA and anti-nuclear autoantibodies are hallmarks of lupus flaring and progression. Mice in the cSiO_2_/CON group exhibited significant increases for both of these autoantibodies in both BALF and plasma compared to VEH/CON (**Fig 10**). Consumption of ↑DHA, ↓SF.ω6, or ↓SF.ω6↑DHA diets significantly reduced cSiO_2_-anti-dsDNA responses in BALF (**Fig 10A**), whereas plasma responses were reduced only in mice fed the ↑DHA diet (**Fig 10B**). Anti-nuclear antibody responses followed similar trends (**Fig 10C and 10D**).

### DHA intake protects against cSiO_2_-induced lesions in the kidney

**Fig 11:**
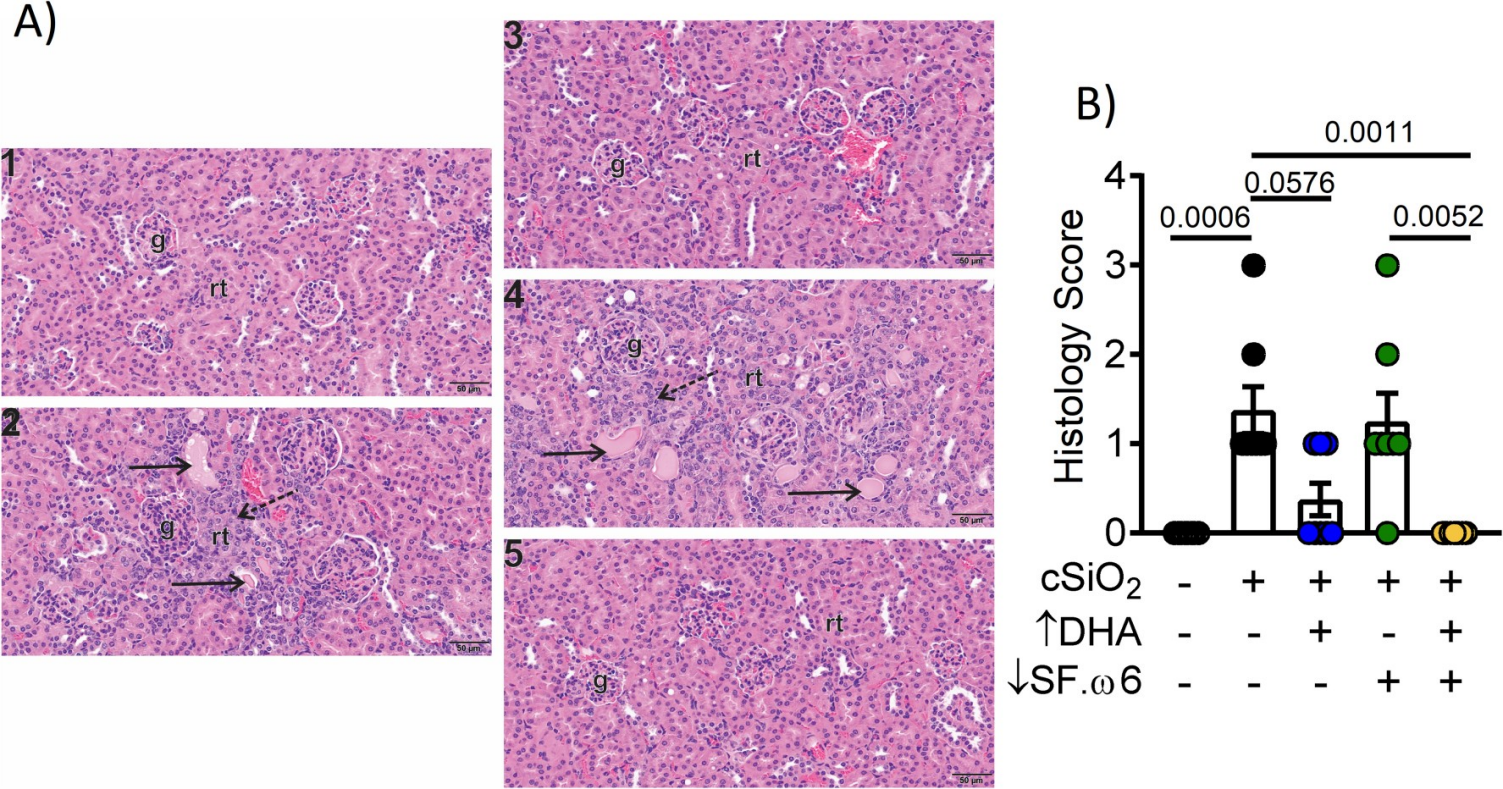
DHA intake inhibits cSiO_2_-induced glomerulonephritis. **(A)** Light photomicrographs of tissue sections from the kidneys of mice intranasally instilled with saline alone (VEH control; 1) or with crystalline silica (cSiO_2_; 2–5). Animals were fed the CON diet (1, 2) or ↑DHA diet (3). Others were fed the ↓SF.ω6 without DHA (4) or with DHA supplementation (↓SF.ω6↑DHA, 5). No renal histopathology was evident in saline-instilled control mice (1) or cSiO_2_-instilled mice fed DHA (3, 5). In contrast, cSiO_2_-instilled mice fed diets without DHA supplementation (2, 4) had renal histopathology characteristic of a membranoproliferative glomerulonephritis characterized by hypercellular glomeruli with thickened mesangial tissue, tubular proteinosis (solid arrows), and tubular epithelial hyperplasia (stippled arrows). **Abbreviations**: g–glomerulus, rt–renal tubules. **(B)** Quantification of renal histology score, based on the following scoring criteria: No proteinosis, normal glomeruli (0); multifocal segmental proliferative glomerulonephritis (1); multifocal segmental proliferative glomerulonephritis and occasional glomerular sclerosis and crescent formation (2); diffuse global segmental proliferative glomerulonephritis (3). Values of p<0.1 are shown, with p<0.05 considered statistically significant.

Naïve female NZBWF1 mice typically display glomerulonephritis around 35 wk of age [52] and die of kidney failure within 1 year. Previous studies in our lab and others have shown that exposure to cSiO_2_ accelerates this phenotype, with nephritis being first observed within 3 months after the final cSiO_2_ instillation (i.e. age 22 wk) in mice fed the AIN-93G diet [16, 29]. While proteinuria was not evident up to experiment termination, histopathological analysis indicated that multifocal segmental proliferative glomerulonephritis was more severe in kidneys of mice after cSiO_2_ instillation fed CON and ↓SF.ω6 compared to mice fed DHA-supplemented diets (**Fig 11A and 11B)**.

## Discussion

**Fig 12:**
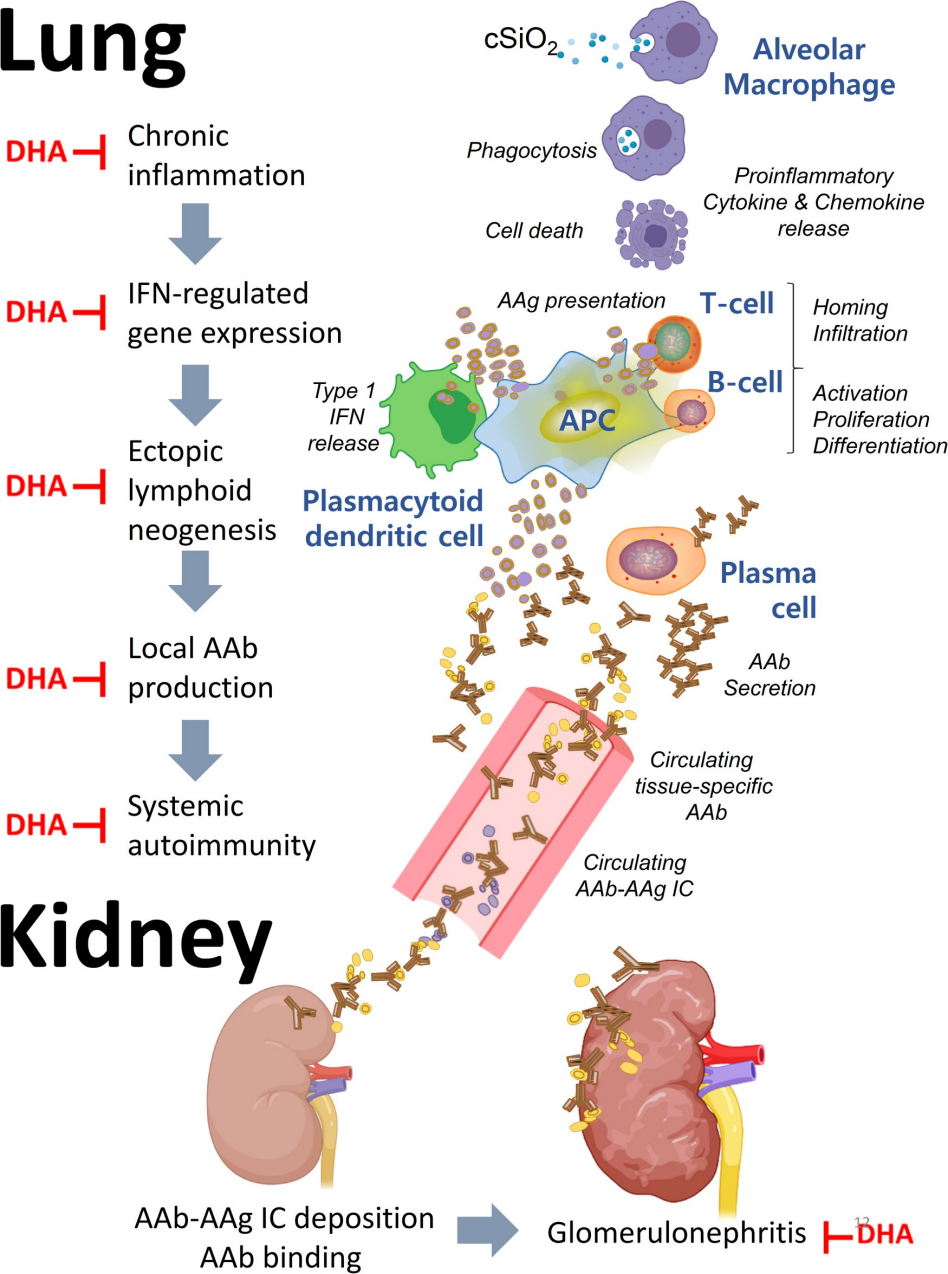
DHA supplementation against the complex background of the Western diet suppresses cSiO2-triggered flaring and progression of lupus in NZBWF1 mice. The results presented here and in other investigations suggest that cSiO_2_ promotes cell death in alveolar macrophages, resulting in the release of proinflammatory cytokines and chemokines that recruit and activate additional immune cells, including T cells and B cells. Accumulation of cellular debris results in uptake and presentation of autoantigens (AAg) to T- and B-cells. Among the cellular debris are host nucleic acids, which stimulate the production of Type I IFN from plasmacytoid dendritic cells. Type I IFN promotes cytokine release, antigen uptake, and maturation of B cells to plasma cells, which produce autoantibodies (AAb) against host antigens both locally and systemically. Upon binding their cognate AAgs, Aabs can form immune complexes that ultimately deposit in the kidney inflicting damage. The red ┴ indicates steps where DHA supplementation has been demonstrated to interfere with this pathway.

The TWD represents typical eating patterns in the U.S, making it highly appropriate for investigating how modulation of dietary lipids affects flaring and progression in preclinical models of lupus. The results presented herein indicate for the first time that translationally relevant DHA supplementation against the complex background of the Western diet is highly effective in protecting against cSiO_2_-triggered IRG expression, cytokine/chemokine release, leukocyte infiltration, ELS neogenesis, autoantibody production in the lungs as well as glomerulonephritis (summarized in Fig 12). While some disease endpoints were modestly attenuated by reducing SFAs and ω-6 PUFAs through increasing the ω-9 PUFA content with olive oil, further DHA supplementation to this diet was required for maximal protection against lupus development. Finally, consistent with the observed effects in the lung, consumption of DHA-amended diets prevented early onset of glomerulonephritis in cSiO_2_-exposed mice. Together, these findings suggest that ω-3 supplementation to a Western diet without substantial diet changes may be protective against lupus flaring.

The observation that DHA can suppress IRG responses is highly significant because IFN signaling has been centrally linked to lupus disease activity in preclinical and clinical studies. In NZBWF1 mice, adenovector-mediated delivery of IFN-α, a major type I IFN, induces the development of lupus [53]. Gonzalez-Quintial, et al. [54] demonstrated in C57Bl6 mice, which are not genetically predisposed to lupus, that early-life virus exposure combined with adult exposure to cSiO_2_ results in the production of lupus-like symptoms, including autoantibody production and glomerulonephritis. Likewise, the IRG signature is closely related to the flaring and pathogenesis of lupus symptoms in human patients [55–57]. Randomized, double-blind, placebo-controlled phase IIb clinical trials indicated that sifalimumab, an anti-IFNα monoclonal antibody [58], and anifrolumab, a type I IFN receptor antagonist [59], positively impacted lupus symptoms. Recently, a large, double-blind, placebo-controlled phase 3 clinical trial (TULIP-2) was finalized and documented that intravenous anifrolumab lowered overall disease activity, reduced skin disease, and enabled oral corticosteroid tapering [60]. Accordingly, the finding here that DHA supplementation of CON and ↓SF.ω6 diets lowered the IRG response is potentially relevant from a translational perspective.

Our analysis of tissue fatty acid content confirmed that the ω-3 PUFA content of the RBC, presented both as the Omega-3 Index and as total ω-3 PUFA, is reflective of ω-3 PUFA levels in the diet [61]. We found here that Omega-3 Indexes for mice fed ↑DHA and ↓SF.ω6↑DHA diets were three times higher than those fed CON or ↓SF.ω6 diets. These trends were consistent with %EPA+DHA levels in the tissues. Alternatively, the effects of feeding ↓SF.ω6 diet on the Omega-3 Index and the %EPA+DHA in tissues were minimal compared to CON-fed animals. This may explain why the ↓SF.ω6 diet provided minimal protection against the cSiO_2_-induced inflammatory response. Additionally, the ↓SF.ω-6 diet had a minor impact on tissue and RBC SFA levels and did not alter levels of long chain ω-6 PUFAs. Though there was a reduction in total ω-6 PUFA levels, this was accomplished primarily by reduction of LA (18:2ω6) rather than ARA (20:4ω6).

Our results suggest that the balance of long chain ω-3 and ω-6 PUFAs in the cell membrane might be critical to promoting inflammation or resolution. One explanation for this observation is that ω-3 and ω-6 PUFAs are substrates for downstream bioactive lipid metabolites. It is well established that lipid metabolites derived from the “arachidonic acid cascade” have primarily inflammatory actions, especially in the case of acute inflammation [62]. Over the last two decades, many metabolites of ω-3 PUFAs have been identified as having anti-inflammatory and pro-resolving properties [63]. A recent study demonstrated that the plasma and RBC levels of ω-3 PUFA were highly correlated with the production of ω-3 PUFA-derived lipid mediators, many of which are involved in the resolution of inflammation. Similarly, supplementation with EPA and DHA led to a decrease in ω-6 PUFAs, namely ARA, as well as decreased ω-6-PUFA derived metabolites [64, 65]. Shifting the membrane composition to favor long chain ω-3 PUFAs rather than ω-6 PUFAs, such as arachidonic acid, may enhance the pro-resolving phenotype promoted by ω-3 PUFA-derived lipid mediators.

Despite a limited number of studies, there is evidence that increasing levels of long chain ω-3 PUFAs in lupus patients leads is protective against inflammation, a process that likely involves the action of bioactive lipid metabolites. A recent study showed strong associations between dietary PUFA intake from fish and the ω-3 status in lupus patients. Further, the RBC ω-3 levels were negatively associated with levels of C-reactive protein [66]. Others have reported that lupus patients had lower levels of plasma ω-3 PUFAs [67] and plasma resolvin D1, an anti-inflammatory metabolite of DHA than healthy controls [68]. Multiple human studies in lupus and other rheumatic diseases have shown decreased disease activity in patients receiving ω-3 supplementation [24], but few studies have investigated the impact of modulating other dietary lipids, such as ω-6 PUFAs and SFAs. To date, there has been no extensive study of the membrane fatty acid content or plasma lipidome of lupus patients. Investigation in this area–both in pre-clinical and clinical settings–is necessary to elucidate potential benefit of ω-3 PUFA supplementation in individuals with lupus.

To summarize, DHA supplementation at a translationally relevant dose was highly effective in preventing cSiO_2_-triggered lupus flaring in NZBWF1 mice fed a Western diet. Future perspectives should include understanding how the TWD may impact the ameliorative effects of lower DHA doses in this model over time and how these responses are influenced by ω-6, SFA, and total fat content. Ultimately, well-designed clinical trials will be needed to confirm the value of ω-3 PUFA supplementation for the prevention and treatment of lupus in humans.

## Supporting information

S1 Table Lung fatty acid content as determined by GLC. (PDF)

S2 Table Liver fatty acid content as determined by GLC. (PDF)

S3 Table Kidney fatty acid content as determined by GLC. (PDF)

S4 Table Spleen fatty acid content as determined by GLC. (PDF)

S5 Table Histopathology severity scores, lungs. (PDF)

S6 Table Urinary protein at 18, 20, and 22 weeks of age. (PDF)

S1 Fig Experimental diets did not affect mouse body weight. (PDF)

S2 Fig Correlation between RBC and tissues for SFA, MUFA, ω-6 PUFA, and ω-3 PUFA. (PDF)

## List of abbreviations

AD: autoimmune disease
AIN: American Institute of Nutrition
ANOVA: analysis of variance
BAFF: B cell activating factor
BALF: bronchoalveolar lavage fluid
Ccl7: chemokine ligand 7
Ccl8: chemokine ligand 8
CON: control diet
cSiO_2_: crystalline silica
Cxcl10: C-X-X motif chemokine 10
DHA: docosahexaenoic acid
DHASCO: microalgal oil containing 40% docosahexaenoic acid
ELS: ectopic lymphoid structure
GLC: gas liquid chromatography
H&E: hematoxylin and eosin
Ifi44: Interferon induced protein 44
Ifit1: interferon-induced protein with tetratricopeptide repeats 1
IFN: interferon
IL-6: interleukin-6
Irf7: interferon regulatory factor 7
IRG: IFN-regulated genes
Isg15: interferon-stimulated gene 15
MCP-1: monocyte chemoattractant protein-1
mTWD: modified total western diet
MUFA: monounsaturated fatty acid
Mx1: MX Dynamin Like GTPase 1
NET: Neutrophil extracellular trap
NHANES: National Health and Nutrition Examination Survey
NZBWF1: New Zealand Black White F1
Oasl1: 2’-5’ oligoadenylate synthetase-like 1
Oas1: 2’-5’ oligoadenylate synthetase 1
Oas2: 2’-5’ oligoadenylate synthetase 2
PASH: Periodic Acid Schiff and hematoxylin
PBS: phosphate buffered saline
Psmb8: proteasome subunit beta type-8
PUFA: polyunsaturated fatty acid
Rsad2: Radical S-Adenosyl Methionine Domain Containing 2
SF: saturated fat
SFA: saturated fatty acid
Siglec1: Sialic Acid-Binding Ig-Like Lectin 1
SLAQ: Systemic Lupus Activity Questionnaire
TNF-α: tumor necrosis factor-alpha
VEH: Vehicle
Zbp1: Z-DNA binding protein-1
