# Supplementary material for: Influence of total western diet on docosahexaenoic acid suppression of silica-triggered lupus flaring in NZBWF1 mice
Source: PLoS One. 2020 May 15;15(5):e0233183. doi: 10.1371/journal.pone.0233183 (PMC7228097; doi:10.1371/journal.pone.0233183)
Supplement: S1 Table — (PDF) [file pone.0233183.s001.pdf]

Table S1: Lung fatty acid content as determined by GLC.

		VEH / CON	$cSiO_2$ / CON	$cSiO_2$ /↑DHA	$cSiO_2$ / ↓SF.ω6	$cSiO_2$ / ↓SF.ω6↑DHA
Common Name	**Chemical Formula**	*(% of total fatty acids)*				
Lauric	**C12:0**	0.07 ± 0.02^{A}	0.03 ± 0.01^{B}	0.04 ± 0.01^{AB}	0.02 ± 0.01^{B}	0.03 ± 0.00^{AB}
Myristic	**C14:0**	1.25 ± 0.17^{A}	2.38 ± 0.40^{BC}	2.74 ± 0.36^{B}	2.21 ± 0.45^{C}	2.10 ± 0.22^{C}
Palmitic	**C16:0**	31.76 ± 1.70^{A}	50.09 ± 5.32^{BC}	52.03 ± 3.45^{BC}	54.07 ± 3.86^{B}	48.55 ± 3.01^{C}
Palmitolaidic	**C16:1ω7t**	0.10 ± 0.02^{A}	0.10 ± 0.01^{A}	0.10 ± 0.01^{A}	0.05 ± 0.01^{B}	0.04 ± 0.01^{B}
Palmitoleic	**C16:1ω7c**	3.21 ± 0.63^{A}	4.02 ± 0.24^{B}	3.94 ± 0.32^{B}	3.07 ± 0.31^{A}	2.94 ± 0.38^{A}
Stearic	**C18:0**	10.25 ± 1.26^{A}	5.21 ± 1.02^{B}	4.82 ± 0.87^{BC}	3.85 ± 0.75^{C}	4.39 ± 0.65^{BC}
Elaidic	**C18:1ω9t**	0.00 ± 0.00	0.00 ± 0.00	0.00 ± 0.00	0.00 ± 0.00	0.00 ± 0.00
Oleic	**C18:1ω9c**	21.01 ± 2.91^{A}	13.81 ± 3.37^{B}	10.43 ± 1.40^{C}	15.88 ± 2.10^{BD}	17.10 ± 1.80^{D}
Linoleic	**C18:2ω6**	7.78 ± 0.75^{A}	5.51 ± 1.04^{B}	5.87 ± 0.66^{B}	2.46 ± 0.30^{C}	3.14 ± 0.40^{C}
Arachidic	**C20:0**	0.09 ± 0.04^{A}	0.03 ± 0.01^{B}	0.04 ± 0.02^{AB}	0.03 ± 0.01^{B}	0.04 ± 0.01^{AB}
Gamma-linolenic	**C18:3ω6**	0.18 ± 0.10^{AB}	0.10 ± 0.02^{A}	0.07 ± 0.01^{B}	0.06 ± 0.02^{BC}	0.04 ± 0.01^{C}
Linolenic	**C18:3ω3**	0.44 ± 0.07^{A}	0.17 ± 0.06^{BC}	0.09 ± 0.03^{B}	0.24 ± 0.08^{C}	0.23 ± 0.08^{C}
Eicosanoic	**C20:1ω9**	0.00 ± 0.00	0.00 ± 0.00	0.00 ± 0.00	0.00 ± 0.00	0.00 ± 0.00
Conjugated Linoleic	**CLA 9c,11t**	0.01 ± 0.03^{A}	0.01 ± 0.02^{A}	0.00 ± 0.00^{A}	0.00 ± 0.01^{A}	0.00 ± 0.01^{A}
Eicosadienoic	**C20:2ω6**	0.28 ± 0.03^{A}	0.16 ± 0.04^{B}	0.13 ± 0.03^{B}	0.07 ± 0.01^{C}	0.08 ± 0.02^{C}
Eicosatrienoic	**C20:3ω9**	0.11 ± 0.03^{A}	0.06 ± 0.02^{BC}	0.03 ± 0.02^{B}	0.10 ± 0.02^{A}	0.07 ± 0.02^{C}
Behenic	**C22:0**	0.05 ± 0.01^{A}	0.04 ± 0.02^{AB}	0.04 ± 0.02^{AB}	0.03 ± 0.01^{B}	0.04 ± 0.01^{AB}
Dihomo-g-linolenic	**C20:3ω6**	0.87 ± 0.13^{A}	0.38 ± 0.08^{BC}	0.44 ± 0.09^{B}	0.28 ± 0.05^{C}	0.37 ± 0.07^{BC}
Arachidonic	**C20:4ω6**	7.70 ± 1.18^{A}	5.83 ± 0.30^{B}	1.63 ± 0.20^{CD}	5.47 ± 0.32^{B}	1.02 ± 0.12^{D}
Eicosapentaenoic	**C20:5ω3**	0.08 ± 0.03^{A}	0.09 ± 0.02^{A}	0.76 ± 0.09^{BC}	0.08 ± 0.02^{A}	0.85 ± 0.08^{C}
Lignoceric	**C24:0**	0.09 ± 0.01^{A}	0.07 ± 0.01^{AB}	0.07 ± 0.01^{AB}	0.05 ± 0.01^{BC}	0.04 ± 0.00^{C}
Adrenic	**C22:4ω6**	2.53 ± 0.39^{A}	1.36 ± 0.31^{B}	0.13 ± 0.04^{C}	1.00 ± 0.19^{B}	0.08 ± 0.02^{D}
Docosapentaenoic ω6	**C22:5ω6**	0.61 ± 0.28^{A}	0.70 ± 0.24^{A}	0.91 ± 0.33^{AB}	0.65 ± 0.22^{A}	1.30 ± 0.36^{B}
Docosapentaenoic ω3	**C22:5ω3**	0.89 ± 0.10^{A}	0.50 ± 0.08^{B}	0.70 ± 0.12^{C}	0.26 ± 0.03^{D}	0.68 ± 0.10^{C}
Docosahexaenoic	**C22:6ω3**	3.04 ± 0.47^{A}	2.40 ± 0.09^{B}	8.88 ± 0.77^{C}	2.12 ± 0.12^{D}	8.87 ± 0.53^{C}
Total SF		44.45 ± 3.07^{A}	58.39 ± 4.96^{B}	60.32 ± 2.94^{B}	60.61 ± 3.58^{B}	55.50 ± 2.61^{B}
Total MUFA		28.89 ± 3.46^{A}	22.51 ± 3.62^{B}	18.67 ± 1.32^{C}	25.75 ± 2.67^{AB}	26.90 ± 1.75^{A}
Total ω-3 PUFA		4.45 ± 0.61^{A}	3.12 ± 0.11^{B}	10.44 ± 0.84^{C}	2.69 ± 0.19^{D}	10.64 ± 0.63^{C}
Total ω-6 PUFA		19.93 ± 1.61^{A}	14.31 ± 1.73^{B}	9.17 ± 1.06^{C}	9.98 ± 0.92^{C}	6.02 ± 0.67^{D}
EPA+DHA		3.12 ± 0.47^{A}	2.49 ± 0.09^{B}	9.65 ± 0.75^{C}	2.20 ± 0.12^{D}	9.72 ± 0.49^{C}

Data presented as mean ± SD. Difference between diets compared by ordinary one-way ANOVA followed by Tukey's multiple comparison. Nonparametric versions of these tests were used when applicable. Unique letters indicate significant differences between groups ($p<0.05$).
