# Supplementary material for: Influence of total western diet on docosahexaenoic acid suppression of silica-triggered lupus flaring in NZBWF1 mice
Source: PLoS One. 2020 May 15;15(5):e0233183. doi: 10.1371/journal.pone.0233183 (PMC7228097; doi:10.1371/journal.pone.0233183)
Supplement: S2 Table — (PDF) [file pone.0233183.s002.pdf]

Table S2: Liver fatty acid content as determined by GLC

		VEH / CON	$cSiO_2$ / CON	$cSiO_2$ /↑DHA	$cSiO_2$ / ↓SF.ω6	$cSiO_2$ / ↓SF.ω6↑DHA
Common Name	**Chemical Formula**	*(% of total fatty acids)*				
Lauric	**C12:0**	0.03 ± 0.01^{AC}	0.04 ± 0.01^{A}	0.04 ± 0.01^{A}	0.02 ± 0.01^{B}	0.02 ± 0.01^{BC}
Myristic	**C14:0**	0.57 ± 0.12^{AC}	0.54 ± 0.10^{AC}	0.69 ± 0.08^{A}	0.31 ± 0.05^{B}	0.54 ± 0.03^{C}
Palmitic	**C16:0**	20.42 ± 0.55^{A}	20.33 ± 0.62^{A}	24.96 ± 1.01^{B}	14.45 ± 0.61^{C}	20.76 ± 1.15^{A}
Palmitolaidic	**C16:1ω7t**	0.06 ± 0.01^{A}	0.06 ± 0.01^{A}	0.06 ± 0.01^{AB}	0.04 ± 0.01^{B}	0.03 ± 0.01^{C}
Palmitoleic	**C16:1ω7c**	2.98 ± 0.48^{AB}	2.48 ± 0.40^{A}	3.28 ± 0.35^{B}	1.70 ± 0.26^{C}	2.58 ± 0.15^{A}
Stearic	**C18:0**	2.73 ± 0.52^{ACD}	3.55 ± 0.52^{B}	3.41 ± 0.80^{BC}	2.08 ± 0.25^{D}	2.10 ± 0.22^{D}
Elaidic	**C18:1ω9t**	0.07 ± 0.02^{A}	0.07 ± 0.01^{A}	0.06 ± 0.01^{A}	0.05 ± 0.02^{A}	0.04 ± 0.02^{A}
Oleic	**C18:1ω9c**	47.01 ± 1.04^{A}	44.79 ± 1.25^{B}	36.04 ± 4.98^{C}	65.50 ± 1.24^{D}	57.44 ± 0.47^{E}
Linoleic	**C18:2ω6**	15.64 ± 1.37^{A}	16.37 ± 1.64^{A}	17.51 ± 3.12^{A}	5.80 ± 0.46^{B}	6.96 ± 0.99^{B}
Arachidic	**C20:0**	0.00 ± 0.00^{A}	0.00 ± 0.00^{A}	0.00 ± 0.00^{A}	0.01 ± 0.02^{A}	0.00 ± 0.00^{A}
Gamma-linolenic	**C18:3ω6**	0.16 ± 0.05^{A}	0.18 ± 0.03^{A}	0.09 ± 0.02^{C}	0.08 ± 0.02^{C}	0.05 ± 0.02^{C}
Linolenic	**C18:3ω3**	0.50 ± 0.09^{A}	0.46 ± 0.10^{A}	1.17 ± 0.14^{B}	1.03 ± 0.14^{BC}	0.90 ± 0.05^{C}
Eicosanoic	**C20:1ω9**	0.49 ± 0.11^{A}	0.52 ± 0.09^{A}	0.03 ± 0.08^{B}	0.00 ± 0.00^{B}	0.00 ± 0.00^{B}
Conjugated Linoleic	**CLA 9c,11t**	0.14 ± 0.02^{AC}	0.13 ± 0.01^{AC}	0.15 ± 0.00^{A}	0.00 ± 0.00^{B}	0.02 ± 0.03^{C}
Eicosadienoic	**C20:2ω6**	0.12 ± 0.02^{A}	0.15 ± 0.02^{B}	0.14 ± 0.03^{AB}	0.05 ± 0.02^{C}	0.06 ± 0.02^{C}
Eicosatrienoic	**C20:3ω9**	0.05 ± 0.01^{AC}	0.07 ± 0.02^{AB}	0.03 ± 0.01^{AC}	0.13 ± 0.02^{B}	0.03 ± 0.01^{C}
Behenic	**C22:0**	0.00 ± 0.00	0.00 ± 0.00	0.00 ± 0.00	0.00 ± 0.00	0.00 ± 0.00
Dihomo-g-linolenic	**C20:3ω6**	0.23 ± 0.02^{ABC}	0.30 ± 0.05^{AC}	0.32 ± 0.06^{AB}	0.19 ± 0.02^{C}	0.15 ± 0.01^{B}
Arachidonic	**C20:4ω6**	1.39 ± 0.19^{A}	2.44 ± 0.57^{B}	0.73 ± 0.17^{C}	1.30 ± 0.19^{A}	0.27 ± 0.03^{D}
Eicosapentaenoic	**C20:5ω3**	0.06 ± 0.01^{A}	0.06 ± 0.02^{A}	0.88 ± 0.29^{B}	0.03 ± 0.01^{C}	0.59 ± 0.10^{B}
Lignoceric	**C24:0**	0.01 ± 0.01^{A}	0.00 ± 0.00^{B}	0.01 ± 0.01^{AB}	0.00 ± 0.00^{B}	0.00 ± 0.00^{B}
Adrenic	**C22:4ω6**	0.06 ± 0.01^{A}	0.09 ± 0.03^{B}	0.04 ± 0.01^{AC}	0.05 ± 0.02^{A}	0.02 ± 0.01^{C}
Docosapentaenoic ω6	**C22:5ω6**	0.32 ± 0.07^{AB}	0.37 ± 0.04^{A}	0.24 ± 0.03^{B}	0.60 ± 0.07^{C}	0.31 ± 0.06^{AB}
Docosapentaenoic ω3	**C22:5ω3**	0.09 ± 0.02^{A}	0.09 ± 0.02^{A}	0.35 ± 0.13^{B}	0.05 ± 0.02^{C}	0.16 ± 0.03^{D}
Docosahexaenoic	**C22:6ω3**	1.02 ± 0.26^{A}	1.55 ± 0.34^{B}	5.07 ± 2.05^{C}	0.73 ± 0.03^{A}	2.08 ± 0.44^{BD}
Total SF		24.10 ± 0.62^{A}	24.82 ± 0.99^{A}	29.44 ± 0.71^{B}	17.06 ± 0.78^{C}	23.60 ± 0.91^{A}
Total MUFA		55.42 ± 1.82^{A}	52.35 ± 1.75^{B}	43.31 ± 6.03^{C}	72.49 ± 1.31^{D}	64.56 ± 0.87^{E}
Total ω-3 PUFA		1.67 ± 0.31^{A}	2.16 ± 0.38^{A}	7.47 ± 2.54^{B}	1.91 ± 0.19^{A}	3.72 ± 0.60^{C}
Total ω-6 PUFA		18.05 ± 1.64^{A}	19.90 ± 1.65^{A}	19.06 ± 3.35^{A}	8.17 ± 0.63^{B}	7.82 ± 1.05^{B}
EPA+DHA		1.08 ± 0.26^{A}	1.60 ± 0.36^{B}	5.95 ± 2.33^{C}	0.75 ± 0.03^{A}	2.67 ± 0.54^{D}

Data presented as mean ± SD. Difference between diets compared by ordinary one-way ANOVA followed by Tukey's multiple comparison. Nonparametric versions of these tests were used when applicable. Unique letters indicate significant differences between groups ($p<0.05$).
