# Supplementary material for: Influence of total western diet on docosahexaenoic acid suppression of silica-triggered lupus flaring in NZBWF1 mice
Source: PLoS One. 2020 May 15;15(5):e0233183. doi: 10.1371/journal.pone.0233183 (PMC7228097; doi:10.1371/journal.pone.0233183)
Supplement: S3 Table — (PDF) [file pone.0233183.s003.pdf]

Table S3: Kidney fatty acid content as determined by GLC

		VEH / CON	cSiO$_2$ / CON	cSiO$_2$ /↑DHA	cSiO$_2$ / ↓SF.ω6	cSiO$_2$ / ↓SF.ω6↑DHA
Common Name	**Chemical Formula**	*(% of total fatty acids)*				
Lauric	**C12:0**	0.12 ± 0.02^{A}	0.11 ± 0.06^{AB}	0.19 ± 0.06^{A}	0.03 ± 0.01^{B}	0.11 ± 0.04^{A}
Myristic	**C14:0**	1.22 ± 0.14^{A}	1.04 ± 0.54^{A}	1.57 ± 0.52^{A}	0.37 ± 0.16^{B}	1.08 ± 0.41^{A}
Palmitic	**C16:0**	19.72 ± 0.51^{A}	18.99 ± 1.30^{A}	22.78 ± 0.95^{B}	15.51 ± 1.18^{C}	18.79 ± 1.52^{A}
Palmitolaidic	**C16:1ω7t**	0.08 ± 0.01^{A}	0.08 ± 0.01^{A}	0.09 ± 0.02^{A}	0.04 ± 0.01^{B}	0.04 ± 0.01^{B}
Palmitoleic	**C16:1ω7c**	5.27 ± 1.49^{A}	3.90 ± 2.05^{AB}	5.21 ± 1.53^{A}	2.09 ± 1.05^{B}	4.29 ± 1.88^{AB}
Stearic	**C18:0**	6.38 ± 1.92^{A}	7.94 ± 5.13^{A}	7.94 ± 2.63^{A}	10.46 ± 4.19^{A}	5.99 ± 3.42^{A}
Elaidic	**C18:1ω9t**	0.09 ± 0.02^{A}	0.08 ± 0.02^{A}	0.07 ± 0.01^{A}	0.04 ± 0.01^{B}	0.04 ± 0.01^{B}
Oleic	**C18:1ω9c**	36.25 ± 4.37^{AB}	33.02 ± 14.35^{AB}	24.46 ± 7.23^{A}	34.15 ± 15.77^{AB}	44.25 ± 12.23^{B}
Linoleic	**C18:2ω6**	14.75 ± 0.60^{A}	14.02 ± 2.13^{A}	15.79 ± 1.49^{A}	7.51 ± 0.05^{B}	8.21 ± 1.20^{B}
Arachidic	**C20:0**	0.06 ± 0.02^{A}	0.09 ± 0.03^{A}	0.06 ± 0.01^{A}	0.08 ± 0.02^{A}	0.06 ± 0.02^{A}
Gamma-linolenic	**C18:3ω6**	0.04 ± 0.01^{A}	0.05 ± 0.02^{A}	0.03 ± 0.01^{AB}	0.04 ± 0.01^{A}	0.02 ± 0.00^{B}
Linolenic	**C18:3ω3**	0.55 ± 0.10^{A}	0.46 ± 0.22^{AC}	0.63 ± 0.15^{A}	0.15 ± 0.07^{B}	0.26 ± 0.09^{BC}
Eicosanoic	**C20:1ω9**	0.26 ± 0.05^{A}	0.28 ± 0.07^{A}	0.16 ± 0.03^{B}	0.35 ± 0.03^{C}	0.23 ± 0.04^{AB}
Conjugated Linoleic	**CLA 9c,11t**	0.15 ± 0.03^{A}	0.14 ± 0.04^{A}	0.14 ± 0.03^{A}	0.05 ± 0.01^{B}	0.05 ± 0.03^{B}
Eicosadienoic	**C20:2ω6**	0.15 ± 0.02^{A}	0.16 ± 0.08^{A}	0.16 ± 0.04^{A}	0.13 ± 0.05^{AB}	0.08 ± 0.03^{B}
Eicosatrienoic	**C20:3ω9**	0.04 ± 0.01^{A}	0.04 ± 0.02^{A}	0.03 ± 0.02^{A}	0.14 ± 0.05^{B}	0.05 ± 0.03^{A}
Behenic	**C22:0**	0.04 ± 0.01^{A}	0.05 ± 0.03^{A}	0.05 ± 0.03^{A}	0.06 ± 0.02^{A}	0.04 ± 0.02^{A}
Dihomo-g-linolenic	**C20:3ω6**	0.31 ± 0.08^{A}	0.40 ± 0.31^{A}	0.50 ± 0.21^{A}	0.64 ± 0.28^{A}	0.37 ± 0.27^{A}
Arachidonic	**C20:4ω6**	6.53 ± 2.56^{AB}	9.11 ± 8.73^{AB}	3.95 ± 2.02^{AB}	15.33 ± 7.30^{A}	2.14 ± 1.72^{A}
Eicosapentaenoic	**C20:5ω3**	0.09 ± 0.02^{A}	0.12 ± 0.11^{A}	2.03 ± 0.98^{B}	0.16 ± 0.07^{A}	2.26 ± 1.73^{B}
Lignoceric	**C24:0**	0.05 ± 0.02^{A}	0.07 ± 0.05^{A}	0.07 ± 0.03^{A}	0.09 ± 0.04^{A}	0.05 ± 0.02^{A}
Adrenic	**C22:4ω6**	0.15 ± 0.05^{AD}	0.24 ± 0.22^{AD}	0.02 ± 0.01^{B}	0.33 ± 0.14^{AC}	0.02 ± 0.01^{BD}
Docosapentaenoic ω6	**C22:5ω6**	0.20 ± 0.04^{A}	0.55 ± 0.55^{AB}	0.55 ± 0.31^{AB}	0.94 ± 0.44^{B}	0.34 ± 0.16^{AB}
Docosapentaenoic ω3	**C22:5ω3**	0.18 ± 0.06^{A}	0.27 ± 0.19^{A}	0.33 ± 0.17^{A}	0.26 ± 0.15^{A}	0.21 ± 0.06^{A}
Docosahexaenoic	**C22:6ω3**	2.84 ± 0.99^{A}	4.12 ± 3.88^{AB}	8.95 ± 4.22^{B}	6.33 ± 2.87^{AB}	7.37 ± 5.17^{AB}
Total SF		27.97 ± 1.67^{A}	28.68 ± 4.35^{AB}	33.07 ± 1.54^{B}	26.87 ± 5.13^{A}	26.33 ± 3.16^{A}
Total MUFA		44.98 ± 4.80^{A}	40.39 ± 16.16^{A}	32.44 ± 8.43^{A}	39.91 ± 16.76^{A}	51.52 ± 13.53^{A}
Total ω-3 PUFA		3.66 ± 1.03^{A}	4.96 ± 3.97^{A}	11.94 ± 5.19^{B}	6.91 ± 3.00^{AB}	10.13 ± 6.93^{AB}
Total ω-6 PUFA		22.12 ± 2.26^{A}	24.53 ± 7.86^{A}	21.00 ± 1.76^{AB}	24.90 ± 8.26^{A}	11.18 ± 3.27^{B}
EPA+DHA		2.93 ± 1.01^{A}	4.24 ± 3.98^{A}	10.98 ± 5.18^{B}	6.94 ± 2.94^{AB}	9.62 ± 6.89^{AB}

Data presented as mean ± SD. Difference between diets compared by ordinary one-way ANOVA followed by Tukey's multiple comparison. Nonparametric versions of these tests were used when applicable. Unique letters indicate significant differences between groups ($p<0.05$).
