# Supplementary material for: Influence of total western diet on docosahexaenoic acid suppression of silica-triggered lupus flaring in NZBWF1 mice
Source: PLoS One. 2020 May 15;15(5):e0233183. doi: 10.1371/journal.pone.0233183 (PMC7228097; doi:10.1371/journal.pone.0233183)
Supplement: S4 Table — (PDF) [file pone.0233183.s004.pdf]

Table S4: Spleen fatty acid content as determined by GLC

		VEH / CON	$cSiO_2$ / CON	$cSiO_2$ /↑DHA	$cSiO_2$ / ↓SF.ω6	$cSiO_2$ / ↓SF.ω6↑DHA
Common Name	**Chemical Formula**	*(% of total fatty acids)*				
Lauric	**C12:0**	0.11 ± 0.03^{A}	0.10 ± 0.03^{A}	0.19 ± 0.07^{B}	0.04 ± 0.01^{C}	0.11 ± 0.04^{AB}
Myristic	**C14:0**	1.18 ± 0.31^{A}	1.17 ± 0.17^{A}	1.85 ± 0.52^{A}	0.61 ± 0.13^{B}	1.22 ± 0.29^{A}
Palmitic	**C16:0**	22.06 ± 2.29^{A}	21.48 ± 1.75^{A}	25.82 ± 1.95^{BC}	19.50 ± 2.89^{A}	23.40 ± 1.98^{C}
Palmitolaidic	**C16:1ω7t**	0.11 ± 0.03^{A}	0.09 ± 0.01^{A}	0.10 ± 0.02^{A}	0.05 ± 0.01^{B}	0.05 ± 0.02^{B}
Palmitoleic	**C16:1ω7c**	5.20 ± 2.10^{A}	4.97 ± 1.14^{A}	5.69 ± 2.08^{A}	3.30 ± 1.46^{A}	4.80 ± 2.18^{A}
Stearic	**C18:0**	7.36 ± 5.00^{A}	6.52 ± 3.01^{A}	7.28 ± 4.02^{A}	8.29 ± 3.95^{A}	7.27 ± 3.27^{A}
Elaidic	**C18:1ω9t**	0.11 ± 0.01^{A}	0.10 ± 0.03^{A}	0.08 ± 0.03^{AB}	0.04 ± 0.02^{B}	0.10 ± 0.06^{A}
Oleic	**C18:1ω9c**	32.81 ± 13.13^{A}	38.21 ± 4.84^{A}	26.20 ± 9.55^{A}	39.67 ± 14.74^{A}	37.40 ± 11.72^{A}
Linoleic	**C18:2ω6**	12.34 ± 2.92^{A}	12.52 ± 1.67^{A}	14.24 ± 2.63^{A}	5.65 ± 0.37^{B}	7.37 ± 0.37^{C}
Arachidic	**C20:0**	0.03 ± 0.03^{A}	0.02 ± 0.04^{A}	0.02 ± 0.03^{A}	0.03 ± 0.03^{A}	0.03 ± 0.04^{A}
Gamma-linolenic	**C18:3ω6**	0.01 ± 0.01^{A}	0.01 ± 0.02^{A}	0.01 ± 0.03^{A}	0.01 ± 0.00^{A}	0.01 ± 0.03^{A}
Linolenic	**C18:3ω3**	0.78 ± 0.13^{A}	0.73 ± 0.11^{A}	0.80 ± 0.14^{A}	0.56 ± 0.12^{AB}	0.33 ± 0.10^{B}
Eicosanoic	**C20:1ω9**	0.00 ± 0.00	0.00 ± 0.00	0.00 ± 0.00	0.00 ± 0.00	0.00 ± 0.00
Conjugated Linoleic	**CLA 9c,11t**	0.08 ± 0.09^{A}	0.11 ± 0.07^{A}	0.09 ± 0.09^{A}	0.02 ± 0.04^{A}	0.01 ± 0.03^{A}
Eicosadienoic	**C20:2ω6**	0.28 ± 0.16^{A}	0.24 ± 0.08^{A}	0.27 ± 0.18^{A}	0.22 ± 0.12^{A}	0.17 ± 0.09^{A}
Eicosatrienoic	**C20:3ω9**	0.04 ± 0.04^{A}	0.06 ± 0.03^{AB}	0.02 ± 0.03^{A}	0.14 ± 0.06^{B}	0.05 ± 0.03^{AB}
Behenic	**C22:0**	0.18 ± 0.17^{A}	0.18 ± 0.09^{A}	0.30 ± 0.31^{A}	0.28 ± 0.24^{A}	0.02 ± 0.02^{A}
Dihomo-g-linolenic	**C20:3ω6**	0.41 ± 0.32^{A}	0.35 ± 0.17^{A}	0.62 ± 0.45^{A}	0.53 ± 0.26^{A}	0.67 ± 0.33^{A}
Arachidonic	**C20:4ω6**	7.21 ± 6.14^{AB}	4.60 ± 3.75^{A}	2.61 ± 2.19^{AB}	9.54 ± 5.28^{A}	1.88 ± 0.98^{B}
Eicosapentaenoic	**C20:5ω3**	0.06 ± 0.05^{A}	0.05 ± 0.02^{A}	0.70 ± 0.51^{AB}	0.06 ± 0.04^{A}	1.19 ± 0.64^{B}
Lignoceric	**C24:0**	0.03 ± 0.05^{A}	0.10 ± 0.08^{AB}	0.09 ± 0.04^{AB}	0.20 ± 0.15^{B}	0.04 ± 0.03^{AB}
Adrenic	**C22:4ω6**	1.11 ± 0.99^{AB}	0.84 ± 0.32^{A}	0.15 ± 0.14^{B}	1.72 ± 0.91^{A}	0.08 ± 0.06^{B}
Docosapentaenoic ω6	**C22:5ω6**	0.70 ± 0.58^{A}	0.56 ± 0.19^{A}	0.98 ± 0.87^{A}	1.08 ± 0.77^{A}	0.34 ± 0.38^{A}
Docosapentaenoic ω3	**C22:5ω3**	0.51 ± 0.42^{A}	0.37 ± 0.16^{A}	0.69 ± 0.54^{A}	0.41 ± 0.19^{A}	0.83 ± 0.46^{A}
Docosahexaenoic	**C22:6ω3**	1.86 ± 1.61^{A}	1.41 ± 0.44^{A}	6.11 ± 4.38^{AB}	2.72 ± 1.42^{AB}	7.66 ± 4.32^{B}
Total SF		31.44 ± 7.13^{A}	30.01 ± 4.74^{A}	36.21 ± 4.50^{A}	29.27 ± 6.99^{A}	32.49 ± 4.85^{A}
Total MUFA		41.77 ± 14.70^{A}	47.29 ± 4.59^{A}	35.17 ± 11.03^{A}	47.14 ± 15.77^{A}	45.83 ± 12.21^{A}
Total ω-3 PUFA		3.21 ± 2.00^{A}	2.54 ± 0.54^{A}	8.29 ± 5.29^{A}	3.74 ± 1.71^{A}	10.01 ± 5.52^{A}
Total ω-6 PUFA		22.05 ± 5.33^{A}	19.85 ± 1.18^{A}	18.89 ± 1.80^{A}	18.75 ± 6.87^{AB}	10.53 ± 1.78^{B}
EPA+DHA		1.92 ± 1.66^{A}	1.45 ± 0.45^{A}	6.81 ± 4.89^{AB}	2.79 ± 1.45^{AB}	8.86 ± 4.96^{B}

Data presented as mean ± SD. Difference between diets compared by ordinary one-way ANOVA followed by Tukey's multiple comparison. Nonparametric versions of these tests were used when applicable. Unique letters indicate significant differences between groups ($p<0.05$).
