# Supplementary material for: Influence of total western diet on docosahexaenoic acid suppression of silica-triggered lupus flaring in NZBWF1 mice
Source: PLoS One. 2020 May 15;15(5):e0233183. doi: 10.1371/journal.pone.0233183 (PMC7228097; doi:10.1371/journal.pone.0233183)
Supplement: S5 Table — (PDF) [file pone.0233183.s005.pdf]

Table S5: Histopathology severity scores, lungs

Experimental Group	LYMPHOID AGGREGATES	ECTOPIC LYMPHOID STRUCTURES	ALVEOLITIS
**VEH / CON**	0 ± 0.1	0 ± 0	0 ± 0.1
**$cSiO_2$ / CON**	3.4 ± 0.3*	2.8 ± 0.4*	3.5 ± 0.3*
**$cSiO_2$ /↑DHA**	1.2 ± 0.3#	0.1 ± 0.1#	2.4 ± 0.2#
**$cSiO_2$ / ↓SF.ω-6**	2.6 ± 0.2	1.5 ± 0.2#	2.4 ± 0.2#
**$cSiO_2$ / ↓SF.ω-6↑DHA**	1.4 ± 0.3#	0 ± 0#	1.1 ± 0.2#

Mice were graded individual for extent of lymphoid aggregation, ectopic lymphoid structure development, and alveolitis (% of total pulmonary tissue examined) as follows: 0 = no changes; 1, minimal (<10%); 2, slight (10-25%); 3, moderate (26-50%); 4, severe (51-75%); 5, very severe (>75%). Data are mean SEM. *Indicates significant difference between VEH/CON and cSiO2/CON groups, as measured by unpaired T test (p<0.05) #Indicates significant difference from cSiO2/Con group, as measured by ordinary one-way ANOVA with Dunnett’s multiple comparison test (p<0.05)
