# Supplementary material for: Influence of total western diet on docosahexaenoic acid suppression of silica-triggered lupus flaring in NZBWF1 mice
Source: PLoS One. 2020 May 15;15(5):e0233183. doi: 10.1371/journal.pone.0233183 (PMC7228097; doi:10.1371/journal.pone.0233183)
Supplement: S6 Table — (PDF) [file pone.0233183.s006.pdf]

Table S6: Urinary protein at 18, 20, and 22 weeks of age.

	Experimental Diets				
	**VEH / CON**	**$cSiO_2$ / CON**	**$cSiO_2$ /↑DHA**	**$cSiO_2$ / ↓SF.ω6**	**$cSiO_2$ / ↓SF.ω6↑DHA**
**Age (weeks)**	*Protein concentration, mg/dL*				
18	61.73 ± 9.77	62.62 ± 12.05	90.00 ± 8.38	58.45 ± 7.20	69.56 ± 7.66
20	56.88 ± 11.88	64.09 ± 9.21	80.24 ± 5.85	76.65 ± 11.08	106.36 ± 31.53
22	233.84 ± 63.18	183.64 ± 38.50	201. 05 ± 24.86	134.45 ± 21.15	214.09 ± 22.68

Data presented as mean ± SE. No significant difference was observed between cSIO2 and any of the groups in week 18, 20, or 22. Mice were euthanized at age 22 weeks
