# Supplementary material for: Influence of total western diet on docosahexaenoic acid suppression of silica-triggered lupus flaring in NZBWF1 mice
Source: PLoS One. 2020 May 15;15(5):e0233183. doi: 10.1371/journal.pone.0233183 (PMC7228097; doi:10.1371/journal.pone.0233183)
Supplement: S1 Fig — (PDF) [file pone.0233183.s007.pdf]

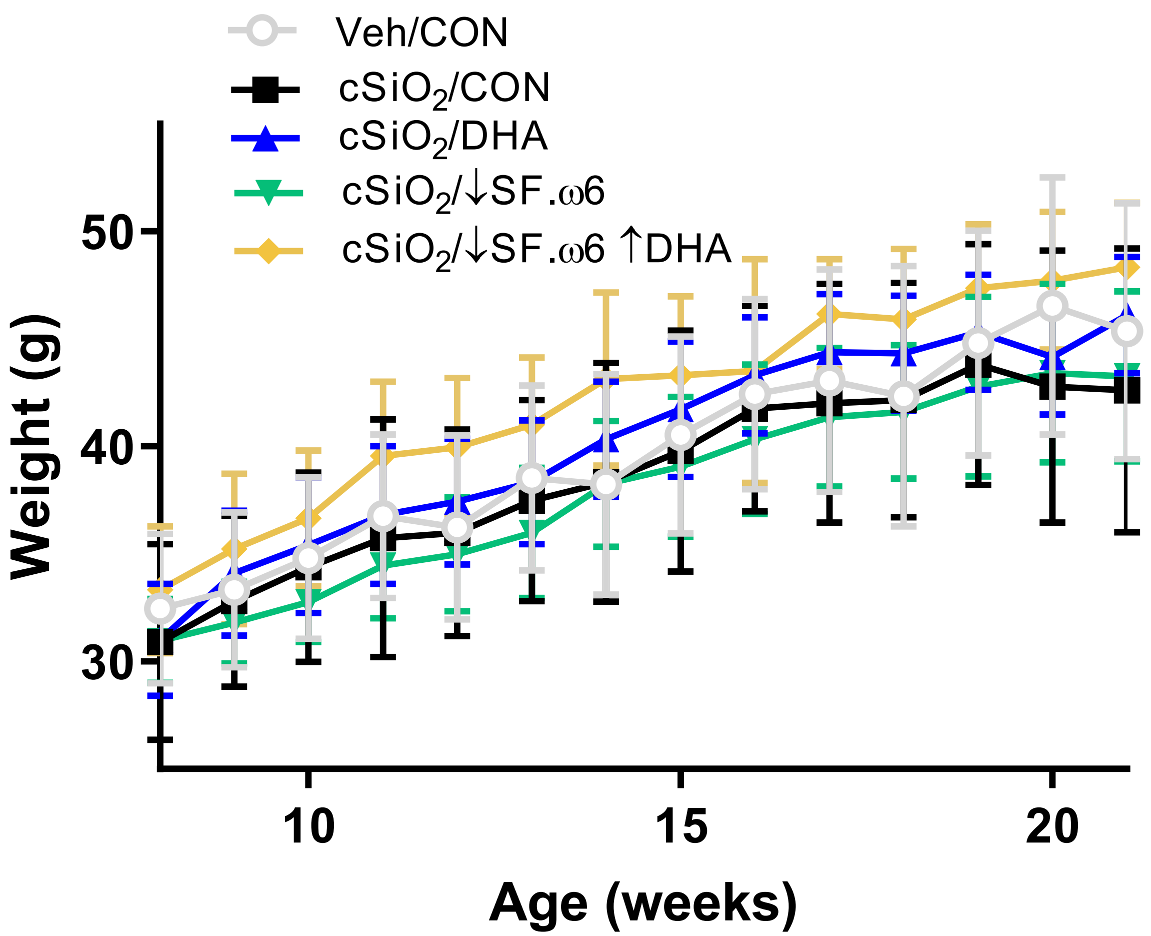

Supplemental Figure S1. Experimental diets did not affect mouse body weight Mice fed isocaloric experimental diets showed no significant difference between body weight at any time point.
