# Supplementary material for: Influence of total western diet on docosahexaenoic acid suppression of silica-triggered lupus flaring in NZBWF1 mice
Source: PLoS One. 2020 May 15;15(5):e0233183. doi: 10.1371/journal.pone.0233183 (PMC7228097; doi:10.1371/journal.pone.0233183)
Supplement: S2 Fig — (PDF) [file pone.0233183.s008.pdf]

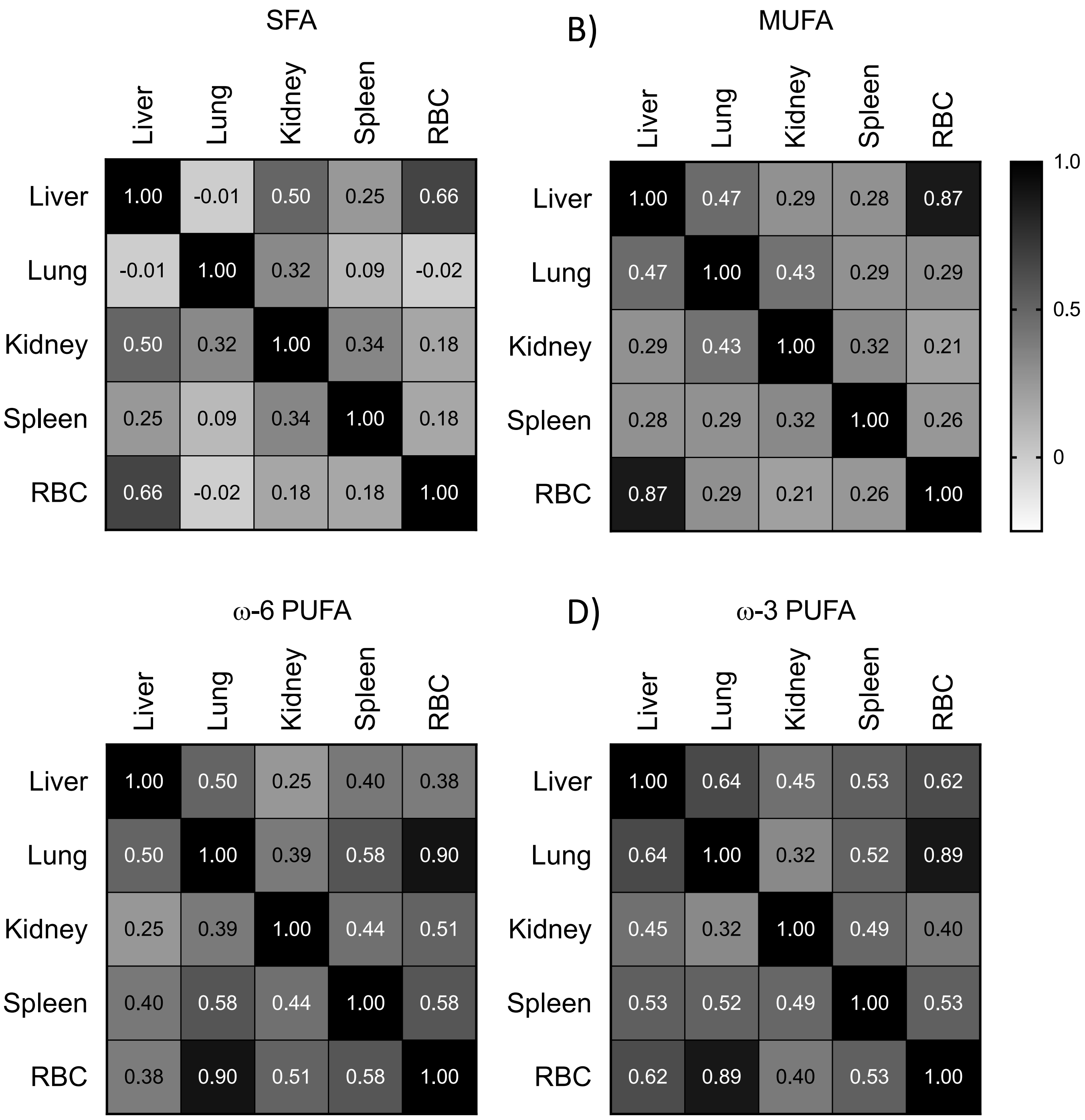

Supplemental Figure S2: Correlation between RBC and tissues for SFA, MUFA, ω-6 PUFA, and ω-3 PUFA. Correlation matrix presenting Pearson's correlation coefficients comparing RBC and tissue levels of SFA **(A)**, MUFA (**B**), ω-6 PUFA (**C**), and ω-3 PUFA (**D**).
